# Glandular Cells of Forest Musk Deer Autonomously Synthesize Sex Steroid Hormones

**DOI:** 10.3390/biology15070583

**Published:** 2026-04-06

**Authors:** Xian An, Xiangyu Han, Jinming Huang, Zexiu Zhang, Zhiyi Lou, Jingyao Hu, Rongzeng Tan, Pengcheng Yang, Xinyue Dou, Habib Bati, Yuetong Zhao, Yele Zhang, Xin Dou, Henghao Zhang, Shuqiang Liu, Congxue Yao

**Affiliations:** 1 College of Nature Conservation, Beijing Forestry University, Beijing 100080, China; axkiki579@bjfu.edu.cn (X.A.); hjymhv233@bjfu.edu.cn (J.H.); 13971877221@163.com (R.T.); yxsdyx18@bjfu.edu.cn (P.Y.); 13836509378@163.com (X.D.); batihabib25@gmail.com (H.B.); z0124m@163.com (Y.Z.); doudn617@bjfu.edu.cn (X.D.); 18600825089@163.com (H.Z.); 2 Beijing Yanshe Biological Technology Co., Ltd., Beijing 102200, China; han_xiangyuwy@163.com; 3 Zhangzhou Pien Tze Huang Pharmaceutical Co., Ltd., Zhangzhou 600436, China; pzhhjm@126.com (J.H.); pzhzzx@126.com (Z.Z.); pzhlzy@zzpzh.com (Z.L.); zyl@zzpzh.com (Y.Z.)

**Keywords:** forest musk deer, musk gland cells, cholesterol, sex steroid hormones, synthesis

## Abstract

The musk gland of male forest musk deer (*Moschus berezovskii*) secretes musk enriched with sex steroid hormones. The testes mainly produce these hormones; however, whether glandular cells can autonomously synthesize them remains unexplored. This study aimed to utilize an in vitro-cultured musk gland cell model to investigate whether musk gland cells possess the capability for autonomous synthesis of sex steroid hormones. We used single-cell RNA sequencing (scRNA-seq), reverse transcription quantitative real-time polymerase chain reaction, and liquid chromatography–mass spectrometry (LC-MS) to verify the steroidogenic potential of musk gland cells. scRNA-seq revealed that during the secretion period, 18 cholesterol and 6 sex steroid hormone biosynthesis genes were significantly expressed in the cells. In vitro experiments demonstrated that these genes were expressed without exogenous cholesterol supplementation. LC-MS analysis confirmed stable synthesis of nine sex steroid hormones. Increasing cholesterol concentration to 20 mg/L significantly upregulated *SRD5A3* and *AKR1D1*, with *AKR1C3* expression showing an upward trend. Elevated cholesterol increased several sex steroid hormone levels: pregnenolone, progesterone, 17α-hydroxypregnenolone, androstenedione, androsterone, and etiocholanolone by 4.12-, 1.46-, 33.42-, 2.06-, 3.11-, and 5.65-fold, respectively. These results collectively indicate that the musk glandular cells can synthesize sex steroid hormones de novo and suggest that cholesterol may regulate their biosynthesis in these cells.

## Simple Summary

Male forest musk deer (*Moschus berezovskii*) secrete musk containing abundant sex steroid hormones, but whether these hormones are synthesized locally within the musk gland remains to be established. Using an in vitro-cultured musk gland cell model, this study investigated the steroidogenic capacity of glandular cells through gene expression analysis and targeted metabolomics. Musk gland cells expressed the complete set of genes involved in cholesterol and sex steroid hormone biosynthesis. In addition, they produced multiple sex steroid hormones in the absence of exogenous hormone supplementation—including pregnenolone, progesterone, and androstenedione—in the absence of exogenous hormone supplementation. Cholesterol supplementation upregulated specific steroidogenic genes and increased the production of several hormones. These findings provide functional evidence that musk gland cells possess autonomous steroidogenic capacity, offering new insights into the biosynthetic origin of musk components and informing future strategies for in vitro musk production.

## 1. Introduction

Forest musk deer (*Moschus berezovskii*), an endangered species of the order Artiodactyla and family Moschidae, are found in China. Male deer have specialized musk glands and sacs that secrete musk, which matures in the sacs during the secretion period [1]. Musk is an important raw material in traditional Chinese medicine and a valuable ingredient in perfumery [2].

Natural musk contains bioactive components, such as macrocyclic ketones, sex steroid hormones, proteins, and peptides. Renowned as the “crown jewel of Chinese medicine,” the Compendium of Materia Medica (Bencao Gangmu) describes the efficacy of musk in restoring consciousness and promoting blood circulation to remove meridian obstructions [3]. Sex steroid hormones are the main component of natural musk, accounting for >5% of its total content [4]. These hormones include cholesterol-derived metabolites, such as androstenedione, testosterone, and androsterone. In addition, they have complex compositions and confer unique pharmacological activities, particularly anti-inflammatory and neuroprotective effects [5].

In male animals, the testes are the primary organs for synthesizing sex steroid hormones [6]. Consequently, in musk deer, sex steroid hormones are believed to be synthesized in the testes and transported through the bloodstream to the musk glands for storage. In addition, serum testosterone levels have been reported to fluctuate synchronously with the musk secretion cycle [7,8,9]. However, recent studies [10], including our own, indicate that sex steroid hormone synthesis genes are also expressed in the musk glands. This suggests that musk gland cells can independently express the necessary enzymes (e.g., *CYP17A1*, *HSD3B*, and *CYP11A1*) for sex steroid hormone synthesis, implying their potential capacity for autonomous synthesis of these hormones. However, due to complex physiological variations in vivo, physiological experiments have been unsuccessful in proving this capability, thereby warranting the need for other evaluation methods.

Previous research on natural musk composition has focused on the following three sex steroid hormones: androsterone, etiocholanolone, and testosterone [3,11,12,13,14,15,16,17,18,19,20]. Cholesterol is the fundamental precursor for all sex steroid hormones [21]. The conversion of cholesterol to pregnenolone is the initial step in all sex steroid hormone syntheses. Pregnenolone is converted to androstenedione via two distinct biosynthetic pathways: the Δ^4^ pathway, involving sequential conversion of pregnenolone to progesterone, 17α-hydroxyprogesterone, and then androstenedione; and the Δ^5^ pathway, in which pregnenolone is converted to 17α-hydroxypregnenolone, then to dehydroepiandrosterone (DHEA), and finally to androstenedione [22], which is further converted by specific enzymes into androsterone, etiocholanolone, and testosterone. Therefore, investigating the changes in cholesterol biosynthesis before and after musk secretion is crucial.

In this study, an in vitro-cultured musk gland cell model was used to investigate whether musk gland cells can synthesize sex steroid hormones autonomously. Furthermore, to investigate the influence of cholesterol on sex steroid hormone synthesis within these cells, cholesterol and nine sex steroid hormones (pregnenolone, progesterone, 17α-hydroxyprogesterone, 17α-hydroxypregnenolone, dehydroepiandrosterone (DHEA), androstenedione, androsterone, etiocholanolone, and testosterone) were measured. The Kyoto Encyclopedia of Genes and Genomes (KEGG) database was used to assess the cholesterol and sex steroid hormone biosynthesis pathways, and multi-omics techniques were used to elucidate their underlying regulating mechanisms.

Eighteen cholesterol biosynthesis genes were identified: *HMGCS1*, *HMGCR*, *MVK*, *PMVK*, *MVD*, *RUSC1*, *FDFT1*, *SQLE*, *LSS*, *CYP51A1*, *TM7SF2*, *MSMO1*, *NSDHL*, *HSD17B7*, *DHCR24*, *EBP*, *SC5D*, and *DHCR7*. Six sex steroid hormone biosynthesis genes were identified: *CYP11A1*, *CYP17A1*, *HSD3B*, *SRD5A3*, *AKR1D1*, and *AKR1C3*. Single-cell RNA sequencing (scRNA-seq) and real-time polymerase chain reaction (RT-qPCR) were used to verify the expression of these 24 genes in musk gland tissue and in an in vitro-cultured musk gland cell line.

## 2. Materials and Methods

### 2.1. Ethics Statement

All animal-related experiments were performed according to the guidelines of the China Council on Animal Care for Wildlife. The Animal Care and Use Committee of Beijing Forestry University approved all animal handling procedures (Permit No: EAWC_BJFU_2022023).

### 2.2. Sample Collection

The musk gland tissues of male forest musk deer during non-secretion and secretion periods were obtained from Beijing Swallow Musk Deer Biotechnology Co., Ltd. (Beijing, China). The musk gland tissues of an 8-year-old adult male in the non-secretion period and a 7-year-old adult male in the secretion period were analyzed to comprehensively investigate the single-cell transcriptome. All animals had succumbed to abscess disease (Table S1). Collected tissues were immediately immersed in ice-cold phosphate-buffered saline (PBS) (Solarbio, Beijing, China) and meticulously cleaned to remove any external blood or debris. Cleaned musk gland tissues were subsequently sectioned into approximately 1 mm^3^ pieces for cell culture.

### 2.3. Single-Cell Library Construction and Sequencing (10× Genomics)

Musk gland tissue was minced into 2–4 mm pieces on ice and degraded using RPMI/enzyme mix (Miltenyi Biotec, Bergisch Gladbach, Germany) on a gentleMACS Octo Dissociator. The resulting cell suspension was filtered through a 70 μm strainer, centrifuged at 300× *g* for 10 min, and treated with Red Blood Cell Lysis Solution (Miltenyi Biotec) for 10 min at 4 °C. After washing with 10 mL 1 × PBS + 0.04% BSA, cell concentration and viability (≥85%) were assessed using CountStar (Shanghai Ruiyu Biotechnology Co., Ltd., Shanghai, China) with acridine orange/propidium iodide (AO/PI) staining. Dead cells were removed using a Dead Cell Removal Kit (Miltenyi Biotec), and viable cells were resuspended in PBS + 0.04% BSA at a concentration of 700–1200 cells/μL for processing on the 10× Genomics Chromium system.

Single-cell RNA-seq libraries were prepared using the Chromium Single Cell 3′ Library & Gel Bead Kit V3.1 (10× Genomics, Inc., Pleasanton, CA, USA), targeting approximately 10,000 input cells. Within the gel bead-in-emulsions, RNA was reverse-transcribed into barcoded cDNA containing unique molecular identifiers. cDNA was amplified using PCR and ligated with sequencing adapters. Libraries were sequenced on an Illumina NovaSeq platform (Berry Genomics, Beijing, China) using a 150 bp paired-end configuration.

Raw BCL files were converted to the FASTQ format using Illumina’s ‘bcl2fastq’. Quality control was conducted, which involved filtering reads containing >3 N bases, >20% low-quality bases (Q-score < 5), or adapter contamination. Data processing was performed using 10× Genomics Cell Ranger software (v7.1; https://www.10xgenomics.com, Accessed on 23 March 2025) for read alignment, barcode/UMI counting, and feature-barcode matrix generation. Given the current lack of a complete, chromosome-level reference genome for forest musk deer, we selected the genome most pertinent to musk gland research as our reference—specifically, the genome constructed by Li et al. [23], which is publicly available in the MuskDB database (http://muskdb.cn/home/, Accessed on 11 May 2025; version v1.0). Alignment statistics are detailed in Supplementary Table S2. Dimensionality reduction, clustering, and gene expression analysis were conducted using default Cell Ranger parameters.

### 2.4. Single-Cell RNA-Seq Data Analysis

Single-cell transcriptome data were loaded using the ‘scanpy’ Python (v3.11) library. Initial data filtering was applied to remove low-quality cells and lowly expressed genes (Figures S1 and S2). Genes detected in <3 cells and cells expressing <200 genes were excluded. Potential doublets were removed using the ‘scanpy.external.pp.scrublet’ function [24]. Further quality control was conducted using the ribosomal gene counts percentage. To precisely remove outlier populations representing low-quality cells or technical artifacts, we established two filtering thresholds: exclusion of cells with a ribosomal gene percentage > 17% (to filter out stressed or low-activity cells) and with >7000 detected genes (to remove potential doublets, as this gene count far exceeds the expected transcriptional complexity range for individual mammalian cells). Thereafter, library size normalization was performed using the ‘scanpy.pp.normalize_total’ function [25]. The log-transformed normalized data matrix was used for downstream analysis.

Dimensionality reduction and unsupervised clustering were performed based on the standard Scanpy workflow. The ‘scanpy.pp.highly_variable_genes’ function [24] to identify the top 4000 highly variable genes (HVGs) for downstream steps. The effects of total UMI counts per cell, mitochondrial gene expression percentage, and ribosomal gene expression percentage were regressed out using the ‘scanpy.pp.regress_out’ function [25]. Each gene was scaled to unit variance using the ‘scanpy.pp.scale’ function with the ‘max_value = 10’ parameter [26].

Following preprocessing, data dimensionality was reduced using principal component analysis. The ‘sc.external.pp.harmony_integrate’ function [27,28] was used with the top 42 principal components to integrate data from samples in different secretion states (secretion vs. non-secretion periods) and to mitigate potential batch effects introduced by the secretion status. UMAP, implemented via the ‘scanpy.tl.umap’ function [29], was subsequently used for further dimensionality reduction of the integrated dataset. Cell clustering was performed using Scanpy’s Leiden algorithm [25].

**Figure 1:**
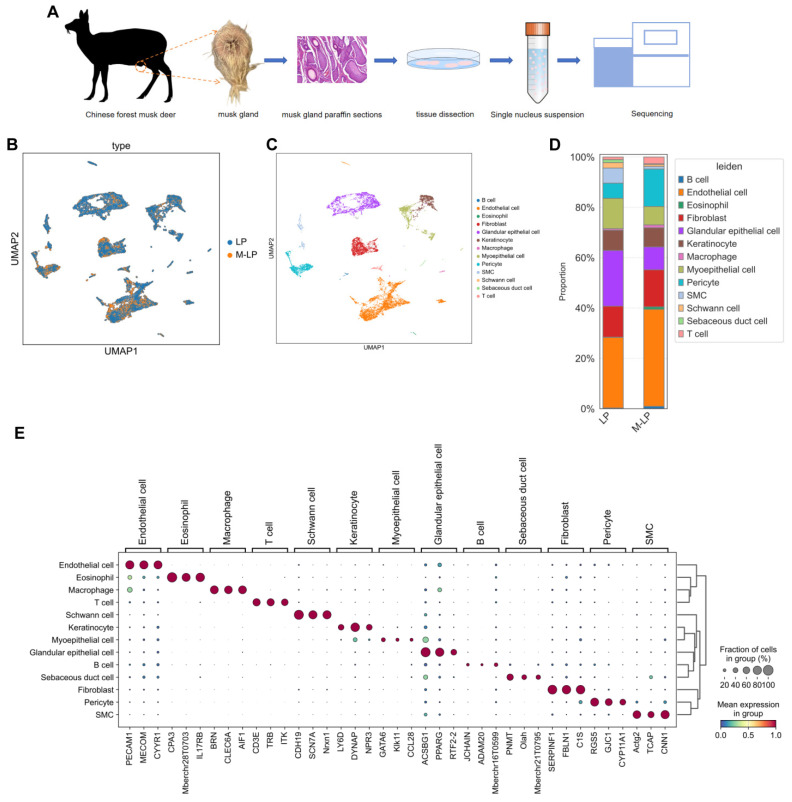
Single-cell transcriptomic analysis of forest musk deer musk gland cells. (**A**) Schematic illustration of the preparation of forest musk deer musk gland tissues for single-cell transcriptome analysis. (**B**) Uniform Manifold Approximation and Projection (UMAP) plot of cells from two different samples (obtained during the secretion and non-secretion periods). Each dot represents a single cell, and dots are color-coded by sample origin. (**C**) UMAP clustering visualization of the 13 major annotated cell types present within the forest musk deer musk gland. (**D**) Proportional composition of the different cell types present in the musk gland before and during the musk secretion period in forest musk deer. (**E**) Dot plot showing the average expression levels of canonical marker genes for the 13 identified cell types.

Marker genes for each cluster were identified using Scanpy’s ‘scanpy.tl.rank_genes_groups’ function [25]. Cell type annotation was performed by comparing these marker genes to known cell type markers (Figure 1E), visualized using the ‘scanpy.pl.dotplot’ function (https://scanpy.readthedocs.io/en/stable/generated/scanpy.pl.dotplot.html, Accessed on 12 May 2025). Provisional annotation of cell clusters was performed by comparing differentially expressed genes with cross-species conserved markers and published musk gland-specific markers (Supplementary Table S3). Differential expression across different cell types or experimental conditions was analyzed using the ‘scanpy.tl.rank_genes_groups’ function [25]. Genes with an adjusted *p* < 0.05 and an absolute log_2_ fold change >1 were considered significantly differentially expressed. To visualize differentially expressed genes, heatmaps were generated using the ‘scanpy.pl.heatmap’ function (https://scanpy.readthedocs.io/en/stable/generated/scanpy.pl.heatmap.html, Accessed on 12 May 2025).

### 2.5. Identification of Genes Related to Cholesterol and Sex Steroid Hormone Synthesis Pathways

Previous studies on natural musk composition were referenced to collate representative sex steroid hormones, which served as the primary targets for this study. The KEGG database (https://www.kegg.jp/, Accessed on 11 May 2024) [30] was used to identify pathways related to cholesterol and sex steroid hormone biosynthesis. The relevant KEGG pathway maps identified were ko00100 (Steroid biosynthesis—primarily cholesterol biosynthesis) and ko00140 (Steroid hormone biosynthesis—primarily sex steroids). These pathway maps presented key enzymes and genes involved in the synthesis and metabolism of our primary target substances. A comprehensive list of genes associated with cholesterol and sex steroid hormone synthesis was extracted from KEGG pathway diagrams. genes directly relevant to the synthesis pathways of our target substances (Table S4). This curated gene list was subsequently used as a reference for further analysis of our single-cell RNA sequencing data to identify cells expressing these genes and investigate their expression patterns across different cell types and conditions.

### 2.6. Primary Forest Musk Deer Cell Culture and Immortalization

**Figure 2:**
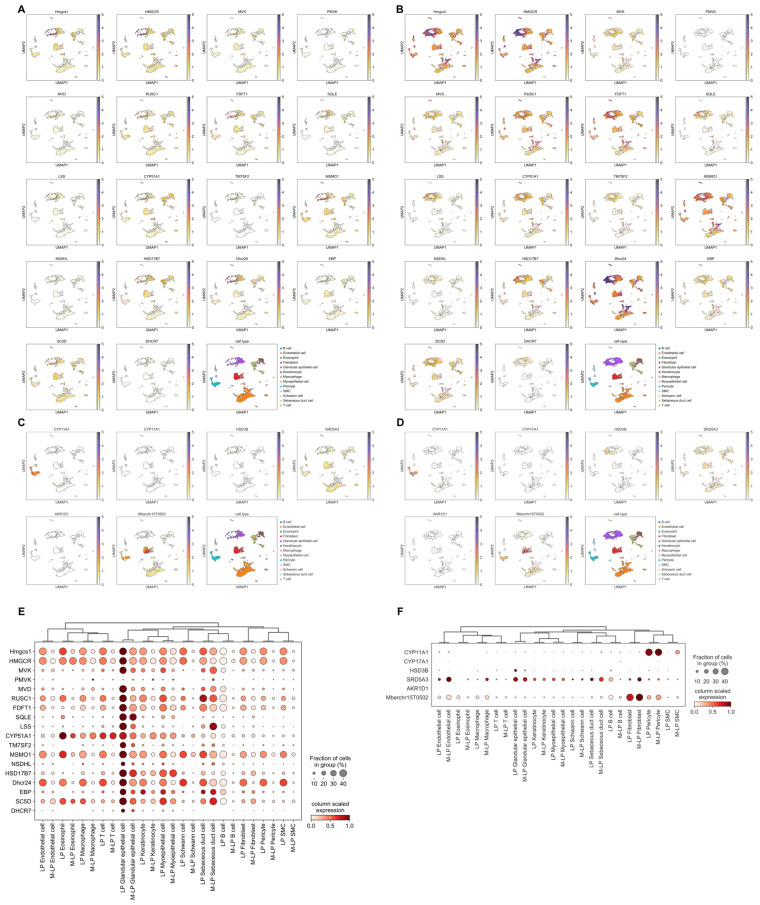
Single-Cell Expression Profiles of Cholesterol and Sex Steroid Hormone Biosynthesis Genes. (**A**) UMAP visualization of the expression of cholesterol biosynthesis-related genes during the secretion period. (**B**) UMAP visualization of the expression of cholesterol biosynthesis-related genes during the non-secretion period. (**C**) UMAP visualization of the expression of sex steroid hormone biosynthesis-related genes during the secretion period. (**D**) UMAP visualization of the expression of sex steroid hormone biosynthesis-related genes during the non-secretion period. (**E**) Dot plot of the same gene sets across the 13 annotated cell types during the secretion period. (**F**) Dot plot of the same gene sets across the 13 annotated cell types during the non-secretion period. Each dot represents a single cell, positioned based on its transcriptomic profile using UMAP dimensionality reduction (axes display arbitrary units). Color intensity indicates the normalized expression level of the aggregated genes within each pathway. Dot size represents the percentage of cells within that cell type expressing the gene. Color scale represents the average normalized expression level among expressing cells.

The collected forest musk deer musk gland tissues were cultured using the tissue explant method. Tissue pieces were finely minced to approximately 1 mm^3^ using ophthalmic scissors. Minced tissue was digested with Collagenase Type I for 20 min, transferred to a 35 mm cell culture dish containing 2 mL culture medium supplemented with 10% fetal bovine serum, 100 U/mL penicillin, and 100 μg/mL streptomycin. The dish was placed in a humidified incubator (37 °C, 5% CO_2_). Cells were passaged after migrating out of the explants and forming a growth halo. Passaging involved trypsinization to detach cells, resuspension in fresh medium, and transfer to a new culture dish. For immortalization, primary musk gland cells were transfected using a viral vector kit (Genomeditech Co., Ltd., Shanghai, China) to encode the SV40 large T antigen gene. Successfully transfected SV40-immortalized musk gland cells were expanded via multiple passages. The forest musk deer glandular cell line established in this study constitutes a mixed cell population derived from primary culture and subsequent SV40-mediated immortalization. This model retains multiple cell types present in the native glandular tissue. The rationale for employing this heterogeneous system was based on our understanding of in vivo glandular organization: single-cell transcriptomic data revealed heterogeneous expression of key genes involved in the sex steroid hormone synthesis pathways across different cell types (Figure 2E,F). This suggests that steroidogenesis in the musk gland may be a multicellular collaborative process. All functional validation experiments were conducted using this mixed cell model.

### 2.7. Forest Musk Deer Cell Treatment Under Different Culture Conditions

Preliminary experiments were conducted to determine the optimal cholesterol concentrations for the cell experiments. Eight cholesterol concentration gradients (0, 10, 20, 30, 40, 50, 60, and 70 mg/L) were prepared using basal medium (Beijing Yanshe Biological Technology Co., Ltd., Beijing, China). To minimize interference from exogenous hormones, all cell cultures were maintained in serum-free medium, thereby allowing the intrinsic biosynthetic capacity of the cells to be clearly discerned.

Based on cell viability and adaptation observed in pre-experiments, the maximum tolerable cholesterol concentration was determined. Consequently, four experimental groups were established for the main study: P1 (basal medium only), D10 (basal medium + 10 mg/L cholesterol), D20 (basal medium + 20 mg/L cholesterol), and A4 (basal medium + agonist). The agonist was provided by our collaborator, Beijing Yanshe Biological Technology Co., Ltd., and is a proprietary compound formulation. Due to pending intellectual property protection, specific details regarding its chemical composition and molecular targets have not been disclosed here. It is a small-molecule compound that modulates HSD3B expression based on preliminary screening data. A cholesterol stock solution was prepared in anhydrous ethanol. All control groups, including the P1 group, were supplemented with an equal volume of ethanol to ensure a final concentration of 0.08%, thereby controlling for any potential solvent effects. The P1 group served as the control, and the D10, D20, and A4 groups were the treatment groups. Cells were cultured in basal medium to approximately 70% confluence before replacing with each experimental group’s respective medium. Three biological replicates were performed for each condition.

### 2.8. Gene Validation via RT-qPCR

mRNA expression levels of co-expressed genes were validated using RT-qPCR. Total RNA was extracted from forest musk deer gland cells subjected to different treatments. First-strand cDNA was synthesized from total RNA using the Fast First-Strand cDNA Synthesis Mix for RT (Albatross Biotech, Guangzhou, China). Primers for cholesterol and sex steroid hormone biosynthesis genes were designed with Primer Premier software (Version 5.0; Premier Biosoft, Palo Alto, CA, USA) and synthesized by Sangon Biotech Co., Ltd. (Beijing, China). All primers were confirmed to yield a single product by melting curve analysis. Primer amplification efficiency was validated using standard curves and ranged between 90% and 110%. For genes with multiple transcript variants (e.g., *HSD3B*), primers were designed within conserved regions to detect total mRNA expression levels. Primer sequences are listed in Table S5. RT-qPCR was performed using Fast Taq SYBR Green qPCR Mix (Albatross Biotech). The RT-qPCR cycling conditions were as follows: initial denaturation at 95 °C for 30 s, followed by 40 cycles of denaturation at 95 °C for 10 s, annealing at 55–65 °C for 10 s, and extension at 72 °C for 30 s. A melting curve analysis step was included with the following conditions: 95 °C for 15 s, 60 °C for 60 s, and 95 °C for 15 s. β-actin (ACTB) served as the endogenous reference gene. Relative mRNA levels were normalized to ACTB levels and calculated using the 2^−ΔΔCT^ method. Data are expressed as the mean ± standard deviation (SD).

### 2.9. LC-MS Metabolomics Analysis

Cells from the P1, D10, D20, and A4 groups were collected after 3 d of treatment. Each cell sample contained at least 1 × 10^7^ cells. A 100 μL sample of initial musk secretion was prepared as the FMA group sample for co-analysis. Samples were flash-frozen in liquid nitrogen for 30 s and stored at −80 °C. Target steroid hormones were quantified using LC-MS. The validated lower limit of quantification (LLOQ) for all target steroids was determined to be 25 pg/mL. Detailed methodology parameters are provided in Supplementary Table S6.

For metabolite extraction, 250 μL of water was added to each sample and then vortexed for 30 s. Thereafter, samples were homogenized using a cell disruptor for 15 min. After homogenization, 50 μL of the homogenate was aliquoted for bicinchoninic acid assay for protein quantification, and the remaining sample was centrifuged at 4 °C and 12,000 rpm (13,800× *g*, rotor radius 8.6 cm) for 15 min. Equal volumes of supernatant from all samples were dried down, and residues were reconstituted in methanol: acetonitrile: water (2:2:1, *v*/*v*/*v*) with isotope-labeled internal standards, and then vortexed for 30 s. Samples were sonicated for 10 min in an ice-water bath and re-centrifuged at 4 °C and 12,000 rpm (13,800× *g*) for 15 min. The resulting supernatant was transferred to autosampler vials for LC-MS analysis; an equal volume of supernatant from each sample was pooled to create a quality control sample, which was also analyzed. Chromatographic separation was performed using a Vanquish UHPLC system (Thermo Fisher Scientific, Waltham, MA, USA) equipped with a Waters ACQUITY UPLC BEH Amide column (2.1 mm × 100 mm, 1.7 μm; Waters Corporation, Milford, CT, USA). Mobile phase A consisted of an aqueous solution of 25 mmol/L ammonium acetate and 25 mmol/L ammonium hydroxide, and mobile phase B was acetonitrile. The gradient elution program was as follows: 0–0.5 min, 95% B; 0.5–7 min, 95–65% B; 7–8 min, 65–40% B; 8–9 min, 40% B; 9–9.1 min, 40–95% B; 9.1–12 min, 95% B. The flow rate was 0.50 mL/min. The autosampler tray temperature was maintained at 4 °C with an injection volume of 2 μL. Mass spectrometric detection was conducted on an Orbitrap Exploris 120 instrument (Thermo Fisher Scientific) with Xcalibur software (v4.4), acquiring MS1 and MS2 spectra. Parameters were as follows: sheath gas flow rate 50 Arb, auxiliary gas flow rate 15 Arb, capillary temperature 320 °C, full MS resolution 60,000, MS/MS resolution 15,000, stepped normalized collision energy 20%, 30%, and 40%, and spray voltage +3.8 kV (positive mode) or −3.4 kV (negative mode).

### 2.10. Statistical Analysis

All quantitative data in this study are presented as the mean ± standard error of the mean (SEM), with the number of independent biological replicates (*n*) explicitly indicated in the corresponding figures. During the experimental design phase, the sample size was not predetermined using any statistical method. The group allocation was not randomized, and investigators were not blinded to group assignment during experimental procedures and outcome assessment. For the analysis of single-cell RNA sequencing data, standard bioinformatic workflows were adhered to. Specifically, cell clustering was performed using the Scanpy toolkit, and differentially expressed genes were identified based on a Bonferroni-adjusted *p*-value < 0.05 and an expression fold change threshold (log_2_ fold change > 1). In the analysis of validation experiment results (including RT-qPCR and LC-MS metabolomics), for comparisons among multiple groups (P1, D10, D20, A4), one-way ANOVA was used, followed by Dunnett’s post hoc test for comparisons against the control group (P1). For pairwise comparisons (e.g., D20 vs. P1 in metabolomics), unpaired two-tailed Student’s *t*-tests were used. Effect sizes (Cohen’s *d*) and 95% CIs were calculated to complement *p*-values. All statistical analyses were conducted using GraphPad Prism software (v10.1.2), with statistical significance defined as follows: ns *p* > 0.05, * *p* ≤ 0.05, ** *p* ≤ 0.01, *** *p* ≤ 0.001, **** *p* ≤ 0.0001.

More detailed information about the samples and statistical parameters is presented in the Supplementary Tables S1–S11.

## 3. Results

### 3.1. Single-Cell Transcriptomics Revealed Metabolic Changes During Musk Secretion

#### 3.1.1. Single-Cell Transcriptomic Profile of Musk Glands

To identify transcriptomic changes that occurred during musk secretion at the single-cell level, we investigated transcriptional differences in the musk gland tissues of forest musk deer (*Moschus berezovskii*) between the secretion and non-secretion periods using the 10× Genomics scRNA-seq system (Figure 1A). Following upstream quality control analysis, transcriptomic profiles from 23,972 cells were obtained for cell type characterization (10,141 cells from the secretion period and 13,831 cells from the non-secretion period; Table S2). An additional quality control step was performed before downstream analysis, resulting in transcriptomic profiles from 23,649 high-quality cells being obtained for cell type characterization. Dimensionality reduction and unsupervised clustering of all cells revealed 21 distinct clusters. Based on differential expression analysis and marker genes identified from the literature (Table S3), 13 major cell types were manually annotated (Figure 1B,C,E; Table S7). These cell types included endothelial cells (characterized by high expression of *Pecam1*, *Mecom*, and *Cyyr1*) [31,32,33], eosinophils (high expression of *Cpa3*, *Tryptase-2* (*Mberchr28T0703*), and *Il17rb*) [34,35], macrophages (high expression of *Brn*, *Clec6a*, and *Aif1*) [36,37], T cells (high expression of *Cd3e*, *Trb*, and *Itk*) [38,39], Schwann cells (high expression of *Cdh19*, *Scn7a*, and *Nrxn1*) [40,41], keratinocytes (high expression of *Ly6d*, *Dynap*, and *Npr3*) [42,43], myoepithelial cells (high expression of *Gata6*, *Klk11*, and *Ccl28*) [44,45], glandular epithelial cells (high expression of *Acsbg1*, *Pparg*, and *Rtf2-2*) [46,47], B cells (high expression of *Jchain*, *Adam20*, and *IGHG* (*Mberchr16T0599*)) [48,49], sebaceous duct cells (high expression of *Pnmt*, *Olah*, and *PAO-LI* (*Mberchr21T0795*)) [50,51], fibroblasts (high expression of *Serpinf1*, *Fbln1*, and *C1s*) [52,53], pericytes (high expression of *Rgs5*, *Gjc1*, and *Cyp11a1*) [54,55] and smooth muscle cells (high expression of *Actg2*, *Tcap*, and *Cnn1*) [56,57].

Cell type abundance analysis revealed that endothelial cells, pericytes, fibroblasts, T cells, B cells, macrophages, and eosinophils exhibited increased proportions within the musk glands during the secretory period compared with those in tissues during the non-secretion period (Figure 1D).

#### 3.1.2. Single-Cell Transcriptomics Revealed Cholesterol and Sex Steroid Hormone Synthesis Genes in Forest Musk Deer Musk Gland Cells

The KEGG database (https://www.kegg.jp/, Accessed on 11 May 2024) was used to identify two pathways related to sex steroid hormone biosynthesis: ko00100 (Steroid biosynthesis-primarily cholesterol biosynthesis) and ko00140 (Steroid hormone biosynthesis-primarily sex steroid hormones). Eighteen genes associated with cholesterol biosynthesis were selected: *HMGCS1*, *HMGCR*, *MVK*, *PMVK*, *MVD*, *RUSC1*, *FDFT1*, *SQLE*, *LSS*, *CYP51A1*, *TM7SF2*, *MSMO1*, *NSDHL*, *HSD17B7*, *DHCR24*, *EBP*, *SC5D*, and *DHCR7*. Additionally, six genes associated with sex steroid hormone biosynthesis were selected: *CYP11A1*, *CYP17A1*, *HSD3B*, *SRD5A3*, *AKR1D1*, and *AKR1C3* (*Mberchr15T0502*).

Single-cell transcriptomic data analysis confirmed the expression of these representative genes within the musk gland tissues of forest musk deer. Their expression distributions across both the musk secretion and non-secretion periods were visualized. This visual elucidated gene expression temporal dynamics and facilitated the identification of potential regulatory mechanisms underlying musk secretion (Figure 2A–D). Furthermore, dot plots were used to delineate differential expression patterns of these genes across various cell types before and after musk secretion onset. This provided a comprehensive overview of gene expression dynamics and highlighted cell type-specific responses to secretion (Figure 2E,F).

During the secretion period, the expression of cholesterol biosynthesis genes was significantly downregulated. In contrast, among the six-sex steroid hormone biosynthesis genes, *CYP11A1*, *CYP17A1*, *SRD5A3*, *AKR1D1*, and *AKR1C3* expression was significantly upregulated, whereas *HSD3B* expression showed a significant increase. The 18-cholesterol biosynthesis-related genes were expressed across all identified cell types. However, their expression levels were markedly higher in glandular epithelial cells compared with those in all other cell types. The expression patterns of the six-sex steroid hormone biosynthesis-related genes exhibited greater cell type specificity: *CYP11A1* was almost exclusively highly expressed in pericytes, with minimal expression in other cell types. *CYP17A1* showed relatively low overall expression across the samples; however, the highest relative expression of *CYP17A1* was observed in B cells. *HSD3B* expression was highest in glandular epithelial cells, whereas its expression in other cell types was low. *SRD5A3* expression was relatively stable across cell types; however, its highest expression was observed in endothelial cells. *AKR1D1* expression was low and only present in some cell types; its highest expression was in glandular epithelial cells. *AKR1C3* (*Mberchr15T0502*) displayed relatively high overall expression; however, its expression was substantially higher in fibroblasts compared with that in any of the other cell types. Complete statistical details of the differential expression analysis for these genes are presented in Supplementary Table S8.

Furthermore, the expression pattern of the steroidogenic acute regulatory (*StAR*) protein, a key initiator of sex steroid hormone synthesis, was analyzed. *StAR* was stably expressed in the musk gland tissues; however, its regulation exhibited remarkable cell type-specificity (Table S7). During the musk secretion period, *StAR* expression was significantly upregulated in pericytes and T cells, while it was sharply downregulated in other cell types, including macrophages and Schwann cells. Downregulation was also observed in endothelial cells, fibroblasts, glandular epithelial cells, keratinocytes, and myoepithelial cells. This complex expression pattern suggests a potentially unique regulatory mechanism for cholesterol transport within the musk glands.

### 3.2. Effects of Different Cholesterol Concentrations on Forest Musk Deer Gland Cells

**Figure 3:**
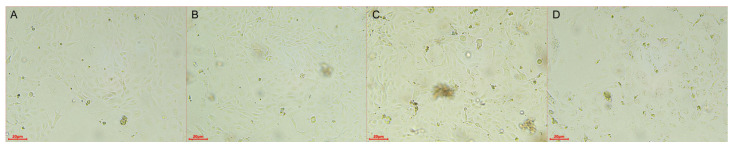
Culture status of forest musk deer glandular cells in the four experimental groups. (**A**) Glandular cells in the P1 group (basal medium). (**B**) Glandular cells in the D10 group (basal medium + 10 mg/L cholesterol). (**C**) Glandular cells in the D20 group (basal medium + 20 mg/L cholesterol). (**D**) Glandular cells in the A4 group (basal medium + agonist).

Forest musk deer glandular cells were cultured in media containing eight cholesterol concentration gradients (0, 10, 20, 30, 40, 50, 60, and 70 mg/L) to determine the maximum concentration tolerable for cell adaptation. The experimental results demonstrated that as cholesterol concentration in the medium increased, the growth rate of the gland cells progressively slowed, accompanied by cellular shrinkage and accelerated detachment. At a cholesterol concentration of 30 mg/L, partial cell death was observed. Consequently, we established 20 mg/L as the maximum cholesterol concentration tolerable for forest musk deer gland cells. Subsequent observation (Figure 3A–D) of the four experimental groups (P1, D10, D20, and A4) revealed distinct growth patterns following medium replacement: In P1 (basal medium), cells exhibited robust growth. Cell death remained low until cells reached 100% confluence and contact inhibition occurred; In D10 (basal medium + 10 mg/L cholesterol), cell growth was relatively stable, accompanied by detachment of a few cells. Cells reached 100% confluence after 2 d of treatment. In D20 (basal medium + 20 mg/L cholesterol), cell growth was marginally slowed, with minor cellular detachment. Cells reached 100% confluence after 2.5 d of treatment, and in the A4 group (basal medium + agonist), cell growth was severely slowed, accompanied by partial cellular detachment. Microscopic examination revealed the presence of lipid droplet-like structures surrounding nuclei in some cells. After 3 d of treatment, cells reached only 80–90% confluence.

### 3.3. RT-qPCR Validation of Cholesterol and Sex Steroid Hormone Synthesis-Related Genes in Forest Musk Deer Gland Cells

**Figure 4:**
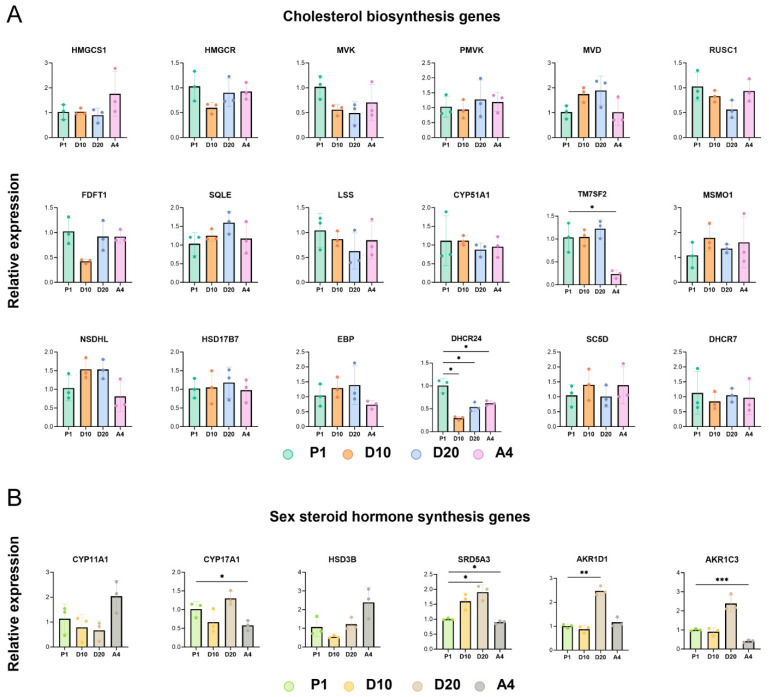
Gene expression levels of 24 target genes under diverse culture conditions. (**A**) mRNA expression levels of 18 cholesterol biosynthesis-related genes in forest musk deer gland cells. (**B**) mRNA expression levels of six sex steroid hormone biosynthesis-related genes in forest musk deer gland cells. The four condition groups were P1 (basal medium), D10 (basal medium + 10 mg/L cholesterol), D20 (basal medium + 20 mg/L cholesterol), and A4 (basal medium + agonist). Data are presented as the mean ± standard deviation (SD) of three independent biological replicates (*n* = 3). Gene expression is normalized to the ACTB housekeeping gene and shown relative to the P1 control group. Statistical significance was determined using one-way ANOVA tests. * *p* < 0.05, ** *p* < 0.01, *** *p* < 0.001.

RT-qPCR analysis validated the mRNA expression levels of the 18 targeted cholesterol and 6 steroidogenic hormone biosynthesis-related genes. All 18 cholesterol biosynthesis genes were expressed in cells cultured under varying cholesterol concentrations and agonist stimulation. However, their mRNA expression variations exhibited no discernible pattern (Figure 4A; Table S9). Compared with that in the P1 group, only *DHCR24* expression was significantly downregulated in both the D10 and D20 groups (*p* = 0.0260 and *p* = 0.0360, respectively). The expression of the other genes showed no significant differences relative to P1; in the A4 group, *TM7SF2* and *DHCR24* expression were significantly downregulated (*p* = 0.0137 and *p* = 0.0141, respectively). All six steroidogenic hormone biosynthesis genes were expressed in all four experimental groups (Figure 4B; Table S9). Relative to that in P1, *SRD5A3* and *AKR1D1* expression was significantly upregulated in the high-cholesterol (20 mg/L) D20 group (*p* = 0.0413 and *p* = 0.0021, respectively). *AKR1C3* expression showed an upward trend under a limited sample size (*n* = 3; *p* = 0.0532). *HSD3B* expression in the agonist-treated A4 group demonstrated an upward trend compared with that in the P1, D10, and D20 groups under a limited sample size (*n* = 3; *p* = 0.0746).

### 3.4. LC-MS Validation Confirmed Autonomous Synthesis of Sex Steroid Hormones in Forest Musk Deer Gland Cells

**Figure 5:**
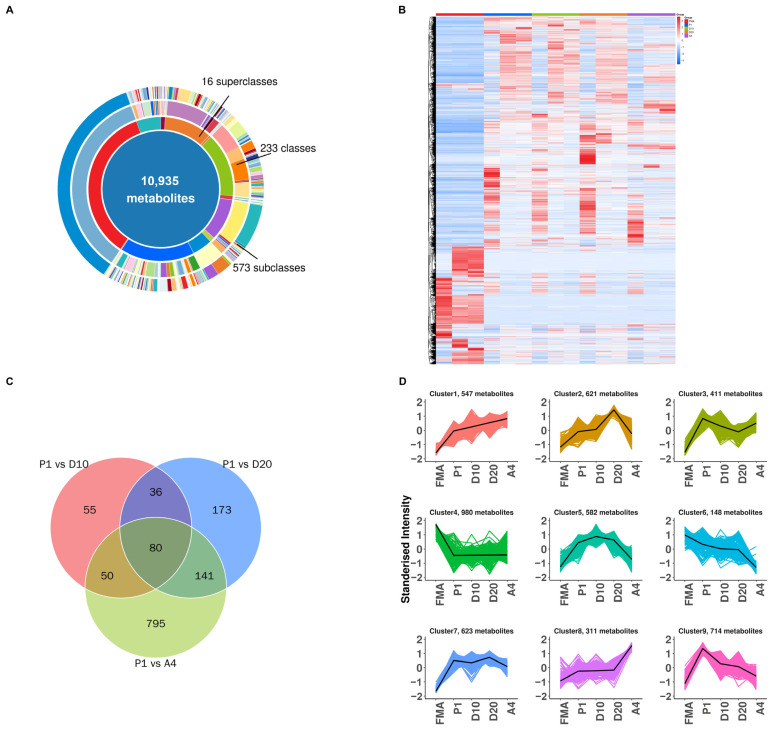
Metabolomics analysis panels. (**A**) Classification of 10,935 identified Level 2 metabolites. (**B**) Clustered heatmap of samples from five groups (FMA, P1, D10, D20, and A4). (**C**) Classification of 10,935 identified Level 2 metabolites. (**D**) Clustered heatmap of samples from five groups (FMA, P1, D10, D20, and A4).

For statistical analysis of the metabolomics data, initial musk secretion (FMA) and the P1 group were designated the controls, whilst D10, D20, and A4 were the treatment groups. In total, there were seven comparison pairs: FMA vs. A4, FMA vs. D10, FMA vs. D20, FMA vs. P1, P1 vs. A4, P1 vs. D10, and P1 vs. D20. After preprocessing, 206,218 features were retained, including 10,935 Level 2 metabolites (putatively annotated compounds), which were subsequently classified (Figure 5A). Data were normalized using clustered heatmap analysis of all five sample groups (FMA, P1, D10, D20, and A4) (Figure 5B). A Venn diagram of the relationships among differential metabolites in P1, D10, D20, and A4 was generated (Figure 5C). Notably, the 100 μL FMA and cell samples (1 × 10^7^ cells/sample) represent distinct biological matrices. Thus, although FMA metabolite profiles informed composition, FMA was excluded from differential comparisons with other groups. The relative levels of all differential metabolites were z-score normalized and subjected to K-means clustering for analysis of the abundance trends (Figure 5D).

Comparative analysis revealed similar metabolite types across the FMA and cell groups (P1/D10/D20/A4); however, their abundance profiles differed. Significant metabolic differences were observed in P1 vs. D10, P1 vs. D20, and P1 vs. A4, with P1 vs. A4 showing the most pronounced changes, indicating the agonist’s strong metabolic influence.

**Figure 6:**
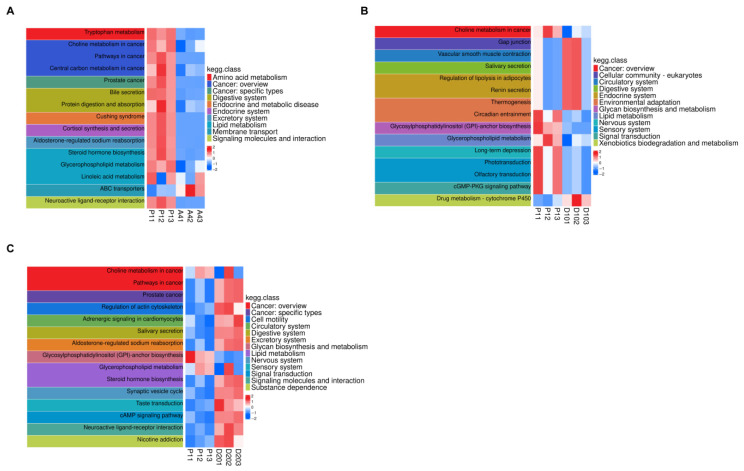
Total abundance distribution of KEGG-annotated differential metabolites across the sample groups. (**A**) Heatmap of summed abundances for KEGG-annotated differential metabolites (P1 vs. A4). (**B**) Heatmap of summed abundances for KEGG-annotated differential metabolites (P1 vs. D10). (**C**) Heatmap of summed abundances for KEGG-annotated differential metabolites (P1 vs. D20).

Pathway heatmaps visualized the summed abundances of KEGG-annotated differential metabolites for P1 vs. A4, P1 vs. D10, and P1 vs. D20 (Figure 6A–C). In P1 vs. A4, among 15 enriched pathways, only ABC transporters exhibited upregulated metabolite abundance in A4; all others (including sex steroid biosynthesis) were downregulated. The upregulation of ABC transporter-related metabolites in the A4 group aligns with a recent study [58], which reported high expression of ABC genes in musk gland cells under agonist stimulation, supporting their potential role in transporting steroidogenic substrates. In P1 vs. D10, gap junction, vascular smooth muscle contraction, salivary secretion, regulation of lipolysis in adipocytes, renin secretion, thermogenesis, and drug metabolism—cytochrome P450—were increased in D10, whereas eight pathways were decreased. In P1 vs. D20, only GPI-anchor biosynthesis was downregulated in D20, whereas 14 pathways (notably sex steroid biosynthesis) were upregulated, confirming that 20 mg/L cholesterol (D20) stimulated hormone synthesis more effectively than 10 mg/L (D10).

**Figure 7:**
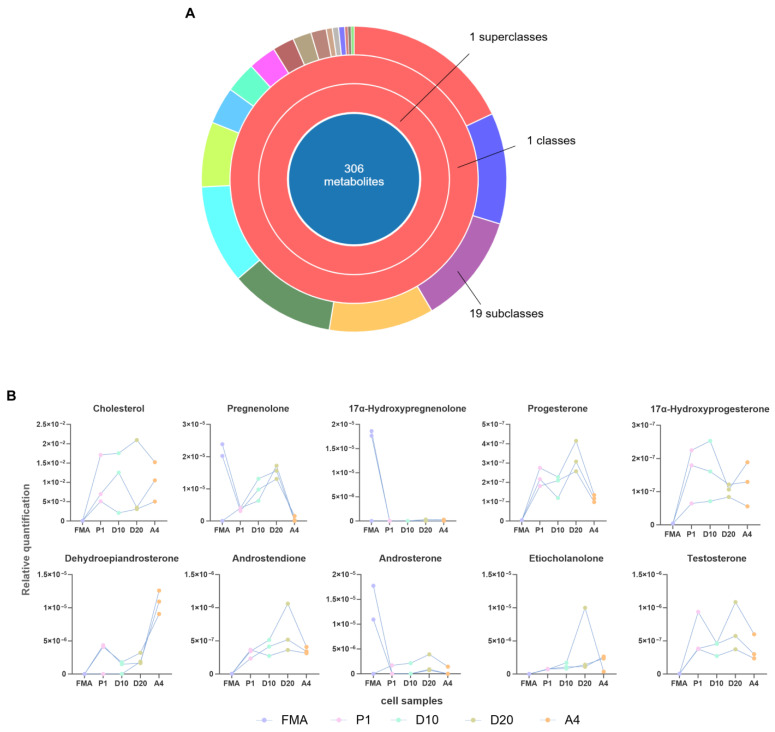
Expression profiles of sex steroid hormones. (**A**) Classification of 306 steroids/derivatives into 19 subclasses. (**B**) Relative abundance of 10 key target substances across the sample groups. *n* = 3/group. The term “Relative quantification” on the vertical axis of (**B**) indicates “Relative abundance (peak area ratio to internal standard)”.

Following metabolite classification and screening across five sample groups, 306 steroids and their derivatives were identified, comprising 19 subcategories: sterol esters, steroidal saponins, steroidal alkaloids, steroidal lactones, pregnane steroids, oxosteroids, androstane steroids, vitamin D and derivatives, physalins and derivatives, hydroxysteroids, sulfated steroids, cycloartenols and derivatives, cucurbitacins, furospirostane steroids and derivatives, stigmastanes and derivatives, azasteroids and derivatives, bile acids, alcohol derivatives, cholestane steroids, and estrane steroids (Figure 7A; Table S10).

We conducted metabolomic analysis of the relative changes in abundance of the 10 key target compounds (cholesterol, pregnenolone, progesterone, 17α-hydroxyprogesterone, 17α-hydroxypregnenolone, DHEA, androstenedione, androsterone, etiocholanolone, and testosterone) among the experimental groups. All 10 compounds were detected in all sample groups, and their relative abundances were presented as line graphs (Figure 7B). Compared to the other four groups, the FMA group exhibited higher relative abundances of pregnenolone, 17α-hydroxypregnenolone, and androsterone. However, the relative levels of the remaining seven compounds were notably low, potentially owing to the secretion residence time of initial musk secretion and preservation methods. Furthermore, with the exception of 17α-hydroxyprogesterone, DHEA, and testosterone, the relative abundances of the other six sex steroid hormones increased with increasing cholesterol concentration in the culture medium, with the D20 group showing the highest levels. Comparison of the D20 and P1 groups (Table S10) revealed a statistically significant increase in pregnenolone (4.12-fold upregulation, *p* = 0.0186) content. In contrast, progesterone, 17α-hydroxypregnenolone, androstenedione, androsterone, and etiocholanolone levels did not significantly change (*p* > 0.05). To assess the magnitude of these non-significant changes between D20 and P1, standardized effect sizes (Cohen’s *d*) and 95% confidence intervals (CI) were calculated. This analysis revealed a 1.46-fold increase in progesterone (*p* > 0.05, *d* = 1.546, 95% CI [−0.278, 3.370]), 33.42-fold increase in 17α-hydroxypregnenolone (*p* > 0.05, *d* = 0.817, 95% CI [−0.849, 2.483]), 2.06-fold increase in androstenedione (*p* > 0.05, *d* = 1.261, 95% CI [−0.491, 3.013]), 3.11-fold increase in androsterone (*p* > 0.05, *d* = 0.872, 95% CI [−0.803, 2.547]), and 5.65-fold increase in etiocholanolone (*p* > 0.05, *d* = 0.960, 95% CI [−0.730, 2.650]) levels.

DHEA production in agonist-treated cells (the A4 group) was 3.86-fold higher than that in the P1 group (*p* = 0.0098), 9.97-fold higher than that in the D10 group (*p* = 0.0011), and 4.86-fold higher than that in the D20 group (*p* = 0.0016).

## 4. Discussion

In this study, scRNA-seq, reverse transcription quantitative RT-qPCR, and LC-MS were utilized to verify the steroidogenic potential of musk gland cells.

scRNA-seq was used to analyze the transcriptomic profiles of musk gland cells from forest musk deer (*Moschus berezovskii*) during the secretion and non-secretion phases. This approach revealed the cellular heterogeneity of the musk gland and identified 13 distinct cell types (Figure 1C). Comprehensive data analysis investigated the expression dynamics of cholesterol and sex steroid hormone synthesis-related genes across the secretion and non-secretion periods, and the heterogeneity of this gene expression among different cell types.

The expression of sex steroid hormone synthesis-related genes was significantly upregulated in musk gland cells during the secretion period. In contrast, genes involved in the synthesis of cholesterol, a fundamental precursor for sex steroid hormones, were significantly downregulated during this period (Figure 2E,F). In steroidogenic cells, cholesterol is a key component of cell membranes and a primary precursor for sex steroid hormones [59]. Thus, considering the role of cholesterol in steroidogenesis, we hypothesize that most cholesterol used by the musk gland is not synthesized locally and is derived from other organs (e.g., the liver). The liver is the central site of cholesterol synthesis in mammals [60], producing approximately 50% of daily total cholesterol [61], and transports it to peripheral tissues via low-density lipoprotein secretion to meet the demands of other organs [62]. Therefore, during the secretion period, liver-synthesized cholesterol may be transported to the musk gland via systemic circulation, supplying essential upstream substrates for sex steroid hormone synthesis.

Although cholesterol synthesis-related genes were broadly expressed across all 13 manually annotated cell types, their expression was the highest in glandular epithelial cells (Figure 2E). In contrast, sex steroid hormone synthesis-related genes exhibited high cell-specific expression; for example, *CYP11A1* was highly expressed only in pericytes, with minimal expression in other cell types (Figure 2F). These findings suggested that one or more cell types within the forest musk deer gland may each possess the intrinsic capacity for sex steroid hormone biosynthesis [63]. The specific cell types involved in this process remain to be identified in our future studies. Moreover, *StAR* expression was notably upregulated in pericytes and T cells but downregulated in other major cell populations, including glandular epithelial cells (Supplementary Table S7). This non-canonical regulatory profile suggests a more complex cholesterol transport network in the musk gland, a non-classical steroidogenic organ, than previously assumed. *StAR* may not serve as the dominant regulatory factor in this context, or its function may be compensated by alternative unidentified mechanisms, which is supported by similar observations in peripheral tissues, such as brain tissue [64].

Preliminary in vitro musk production studies in China established culture conditions for musk gland cells [65] and generated immortalized musk gland cell lines [66,67]. Building on this, we developed an immortalized forest musk deer cell line via SV40 viral transfection. Although cell purity was not quantitatively assessed by flow cytometry, this cellular model successfully expressed the full complement of genes essential for the sex steroid hormone synthesis pathway (Figure 4) and consistently produced multiple sex steroid hormones (Figure 7B). Future studies could use cell sorting techniques to further explore the specific contributions of distinct cellular subpopulations to sex steroid hormone production.

RT-qPCR analysis of the expression of genes associated with steroidogenic hormone and cholesterol synthesis pathways in primary musk gland cells demonstrated stable constitutive expressions of both cholesterol biosynthesis and steroidogenic hormone biosynthesis genes (Figure 4A,B); however, most of the 18 cholesterol biosynthesis genes exhibited no significant alterations in their expression levels following changes in cholesterol concentration or agonist exposure (Figure 4A; Table S9), indicating that neither the exogenous cholesterol supplementation nor agonist treatment substantially modulated cholesterol synthesis gene expression in musk gland cells. Consequently, these findings suggested that during the musk secretion period, cholesterol synthesis gene transcriptional regulation in musk gland cells may operate via alternative mechanisms independent of feedback control by end-product cholesterol accumulation.

In the high-cholesterol D20 group, *SRD5A3* and *AKR1D1* expression was significantly upregulated, and *AKR1C3* expression exhibited an upward regulatory trend with limited samples (*n* = 3; Figure 4B; Table S9). These findings indicated that *SRD5A3*, *AKR1D1*, and *AKR1C3* may play pivotal roles in the steroidogenic pathway, where their upregulation likely enhances the biosynthetic efficiency of sex steroid hormones in musk gland cells.

Metabolomic profiling of cells under different culture conditions confirmed that musk gland cells can synthesize sex steroid hormones, whilst enabling rapid musk secretion. Musk gland cells synthesized these hormones even without exogenous cholesterol (Figure 7B). High-concentration cholesterol (20 mg/L, D20) substantially increased hormone production, whereas low-concentration cholesterol (10 mg/L, D10) had minimal effects. In addition, agonist treatment (group A4) specifically elevated DHEA production.

Leydig cells cultured without exogenous cholesterol use intracellular cholesterol stores for steroidogenesis, with prolonged human chorionic gonadotropin (hCG) stimulation depleting these stores and upregulating cholesterol synthesis. Exogenous cholesterol supplementation under such conditions can restore sex steroid hormone production [68]. Another study found that hypocholesterolemia did not alter cholesterol content or steroidogenesis in Leydig tumor cells; hCG stimulation enhanced steroidogenesis and cholesterol synthesis without changing intracellular cholesterol levels, indicating reliance of these cells on de novo cholesterol synthesis to support steroidogenesis. These cells maintain sex steroid hormone production under limited exogenous cholesterol by upregulating intracellular synthesis [69], and highlighting their autonomous regulatory capacity. Our RT-qPCR data showed stable cholesterol and sex steroid synthesis gene expressions in group P1 (no exogenous cholesterol), suggesting musk gland cells may similarly autonomously regulate synthesis, initially using stored cholesterol and subsequently boosting steroidogenesis via de novo synthesis or exogenous uptake.

Metabolomic analysis confirmed the expression of all nine targeted sex steroid hormones in the P1 group, demonstrating the intrinsic capacity of in vitro musk gland cells for autonomous sex steroid hormone synthesis. Furthermore, 20 mg/L cholesterol (D20 group) supplementation promoted substantial upregulation of six sex steroid hormones (Figure 7B, Table S11), with pregnenolone levels exhibiting a 4.12-fold increase compared with those in the P1 group. In contrast, progesterone, 17α-hydroxypregnenolone, androstenedione, androsterone, and etiocholanolone levels did not significantly change. To comprehensively assess the biological relevance of these non-significant changes, standardized effect sizes (Cohen’s *d*) and 95% confidence intervals (CI) were employed [69]. The D20 group showed a 1.46-fold increase in progesterone, 33.42-fold increase in 17α-hydroxypregnenolone, 2.06-fold increase in androstenedione, 3.11-fold increase in androsterone, and a 5.65-fold increase in etiocholanolone levels compared with those in P1. Notably, owing to the small sample size (*n* = 3), the effect size CIs were exceptionally wide; therefore, transparency is needed regarding their reporting; however, interpretation should focus on effect direction rather than the absolute magnitude. The standardized effect sizes and CIs in this study complement *p*-values in assessing how varying cholesterol concentrations and agonist A4 modulate sex steroid hormone output in musk gland cells. Statistical significance (*p*-value) alone does not fully capture effect magnitude or biological importance [70,71,72], as observed increases, even without statistical significance, may hold biological relevance. Combining standardized effect sizes with CIs provides a more comprehensive evaluation of the biological significance of metabolite changes [68]. Cohen’s *d* values of 0.2, 0.5, and 0.8 correspond to small, medium, and large effect sizes, respectively [73]. Notably, all five non-significantly changed hormones in the D20 vs. P1 comparison had Cohen’s *d* > 0.8, collectively suggesting biologically meaningful increases in the D20 group. These findings support the inference that elevated cholesterol concentration may promote sex steroid hormone biosynthesis.

Agonist treatment significantly upregulated DHEA production in musk gland cells. Specifically, the A4 group exhibited 3.86-, 9.97-, and 4.86-fold increases in DHEA content compared with those in the P1, D10, and D20 groups. Subsequent RT-qPCR analysis revealed an increase in *HSD3B* expression in the agonist-treated A4 group compared with that in both the D10 and D20 groups (*n* = 3, *p* = 0.0746) (Figure 4B, Table S9). *HSD3B* regulates DHEA biosynthesis, and insulin, insulin-like growth factor I, epidermal growth factor, transforming growth factor β1, and vitamin A can promote its expression [74,75,76]. Therefore, the agonist is thought to contain components that modulate *HSD3B* expression and, consequently, DHEA synthesis.

This study examined whether forest musk deer gland cells autonomously produce sex steroid hormones. In vivo single-cell transcriptomics revealed how the physiological activities during the secretion period drive hormone production. Combined with in vitro cell culture and metabolomics, these findings elucidated the molecular mechanisms underlying autonomous steroidogenesis in the musk gland, providing a theoretical foundation for in vitro musk production. Our results demonstrate that musk gland epithelial cells possess complete steroidogenic capability. Exogenous cholesterol supplementation may enhance sex steroid hormone production, offering a novel strategy for in vitro musk synthesis via cell culture.

## 5. Limitations of the Study

Despite these promising findings, several limitations of this study should be acknowledged. The single-cell transcriptomic data came from only one animal per time point, constrained by the difficulty of sampling endangered forest musk deer. While this limited our ability to account for individual variation, the data still provided useful hypotheses that we followed up with in vitro experiments. The agonist used in cell culture came from a collaborator, and its exact composition could not be disclosed due to intellectual property concerns, which means we could only observe its effects without fully explaining the mechanism. Our in vitro model used mixed cell populations, even though the single-cell data hinted that different cell types might work together during steroidogenesis; teasing apart each cell type’s role will need to wait for future studies. For genes with multiple transcript variants, we designed RT-qPCR primers to capture overall expression rather than isoform-specific patterns, which may have masked some differences. The 20 mg/L cholesterol concentration was chosen based on how cells looked under the microscope rather than quantitative viability data. Although the increases in progesterone, 17α-hydroxypregnenolone, androstenedione, androsterone, and etiocholanolone in the D20 group did not reach statistical significance (*p* > 0.05), the large effect sizes (Cohen’s *d* > 0.8) suggest potential biological relevance that warrants validation in future studies with larger sample sizes. These limitations point clearly toward our next steps, and future work will address these issues with improved experimental designs.

## 6. Conclusions

This study demonstrates that forest musk deer musk gland cells possess the capacity for autonomous sex steroid hormone biosynthesis. In vitro-cultured glandular cells expressed the complete gene sets for cholesterol and steroid hormone biosynthesis and produced multiple sex steroid hormones without exogenous hormone input, confirming the self-sufficiency of the steroidogenic pathway within the musk gland. Single-cell transcriptomic analysis revealed that steroidogenic genes exhibit distinct expression patterns across different cell types, suggesting that one or more cell subtypes may possess the capacity for sex steroid hormone synthesis. Exogenous cholesterol supplementation upregulated selected steroidogenic genes and enhanced the production of several hormones, indicating that substrate availability may modulate biosynthetic output. These findings indicate that the forest musk deer musk gland is an independent site of sex steroid hormone biosynthesis and provide a foundation for further mechanistic studies on musk formation and the development of cell-based approaches for musk production.

## Data Availability

All processed data supporting the findings of this study—including single-cell gene expression matrices, cell metadata, and processed metabolomics results—together with the primary analysis code used to generate the key figures, have been publicly deposited in the Mendeley Database (https://doi.org/10.17632/kz2ykcvg5h.1) and Zenodo (https://doi.org/10.5281/zenodo.18102405).

## Supplementary Materials

The following supporting information can be downloaded at: https://www.mdpi.com/article/10.3390/biology15070583/s1, Figure S1: Pre-QC Violin Plots; Figure S2: Post-QC Violin Plots; Table S1: Animals sample information; Table S2: Cell QC.summary; Table S3: Cell Maker; Table S4: KEGG-cholesterol synthesis genes and sex steroid hormone synthesis genes; Table S5: Primer; Table S6: List of Key Detection Targets with Assayed Ranges; Table S7: Gene Expression Prevalence Across Cells; Table S8: Analysis of expression differences of 24 genes in 13 cell types; Table S9: RT-qPCR Statistical Supplement; Table S10: Relative Abundance of Sex Steroid Hormones; Table S11: Sex Steroid Hormone Levels Statistical Supplement.

## Abbreviations

The following abbreviations are used in this manuscript:

scRNA-seq	Single-cell RNA sequencing
RT-qPCR	Real-time polymerase chain reaction
LC-MS	Liquid chromatography–mass spectrometry
DHEA	Dehydroepiandrosterone
KEGG	Kyoto Encyclopedia of Genes and Genomes
PBS	Phosphate-buffered saline
BSA	Bovine serum albumin
AO/PI	Acridine orange/propidium iodide
UMI	Unique molecular identifier
HVGs	Highly variable genes
UMAP	Uniform Manifold Approximation and Projection
ACTB	β-actin
SD	Standard deviation
hCG	Human chorionic gonadotropin
