# Supplementary figures and images for: Glandular Cells of Forest Musk Deer Autonomously Synthesize Sex Steroid Hormones
Source: Biology (Basel). 2026 Apr 6;15(7):583. doi: 10.3390/biology15070583 (PMC13071994; doi:10.3390/biology15070583)

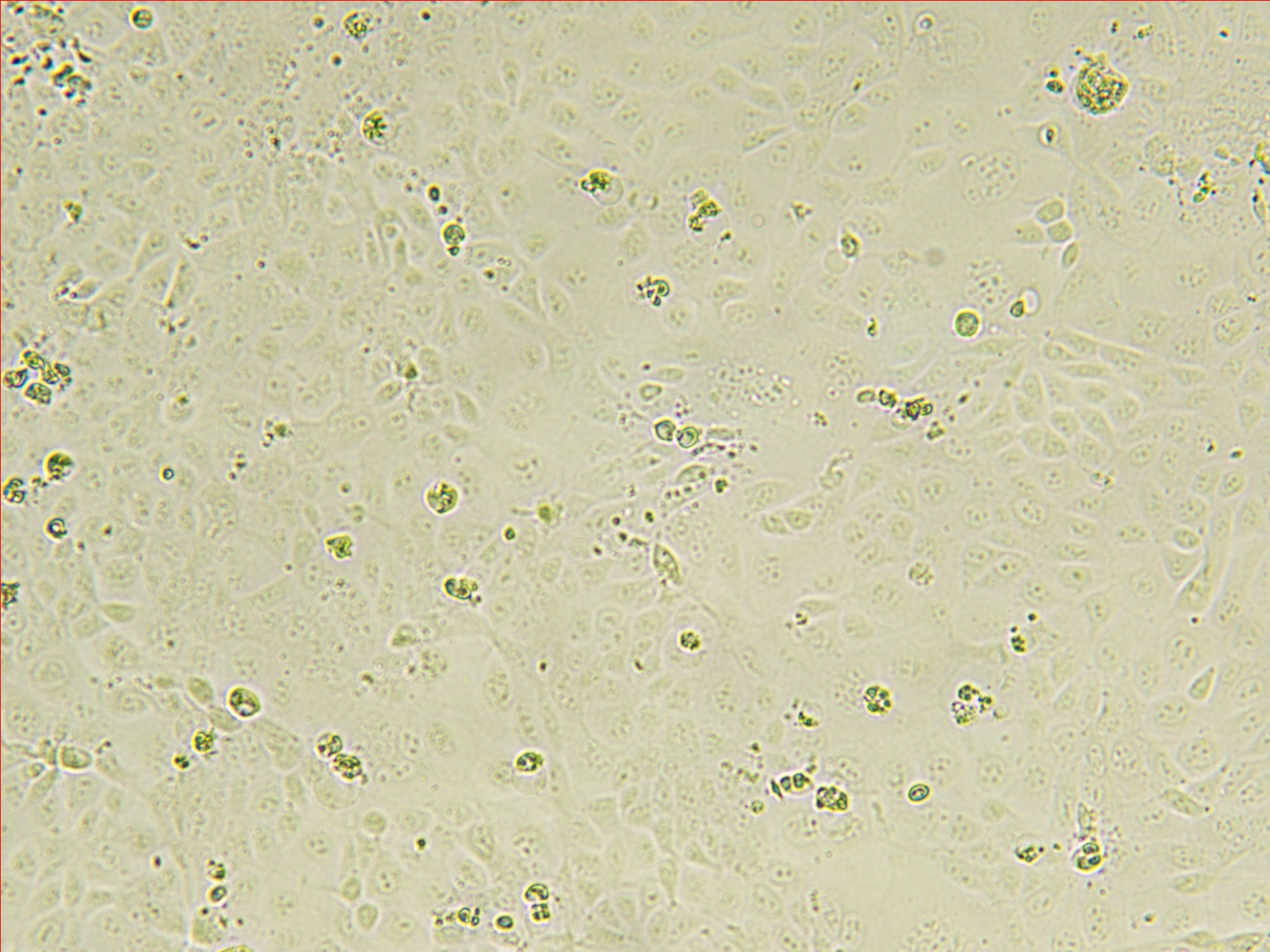

Supplement: Supplementary file 1 [file biology-15-00583-s001.zip › 0 mgL cholesterol-10×.jpg]

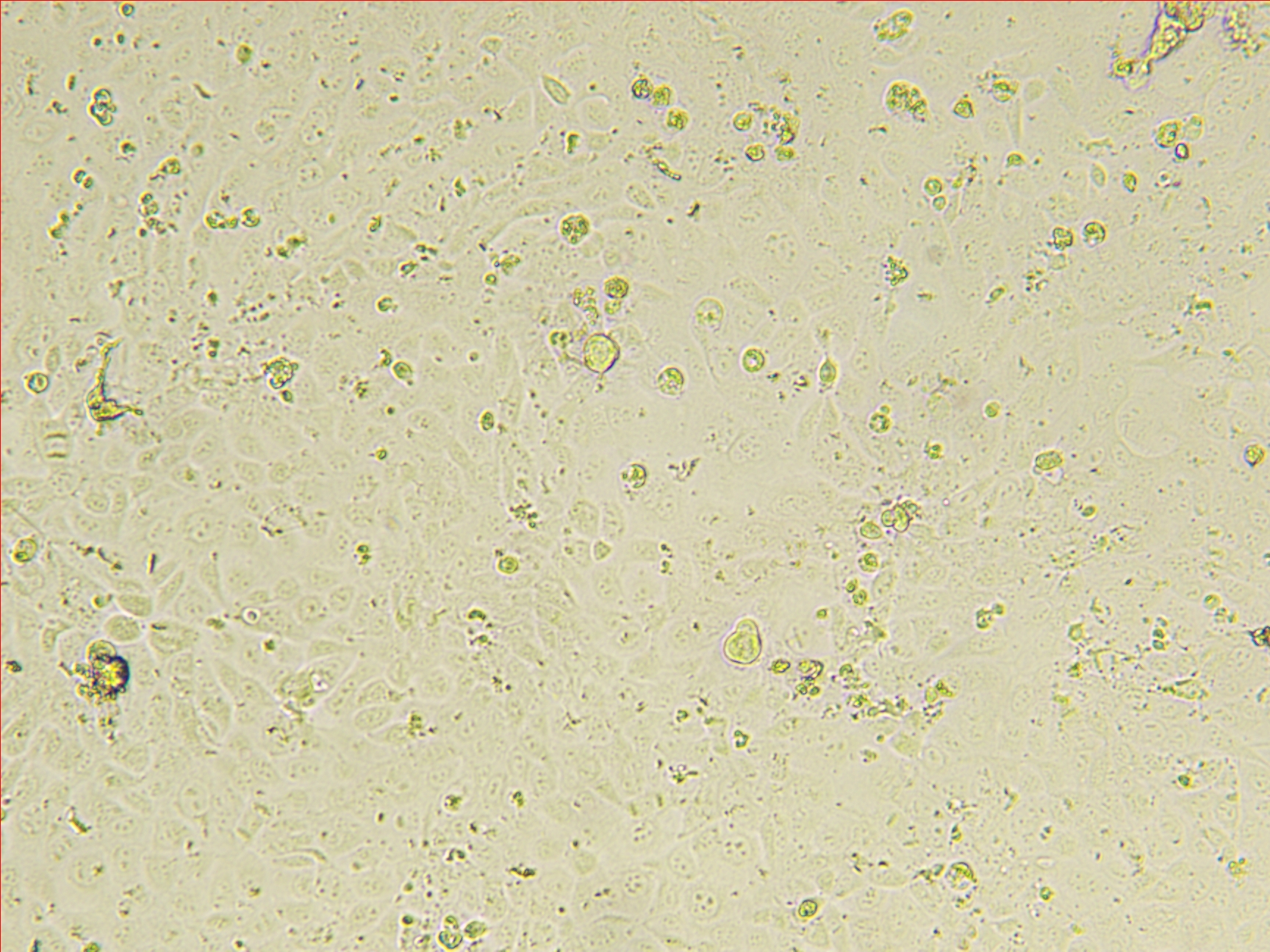

Supplement: Supplementary file 1 [file biology-15-00583-s001.zip › 10 mgL cholesterol-10×.jpg]

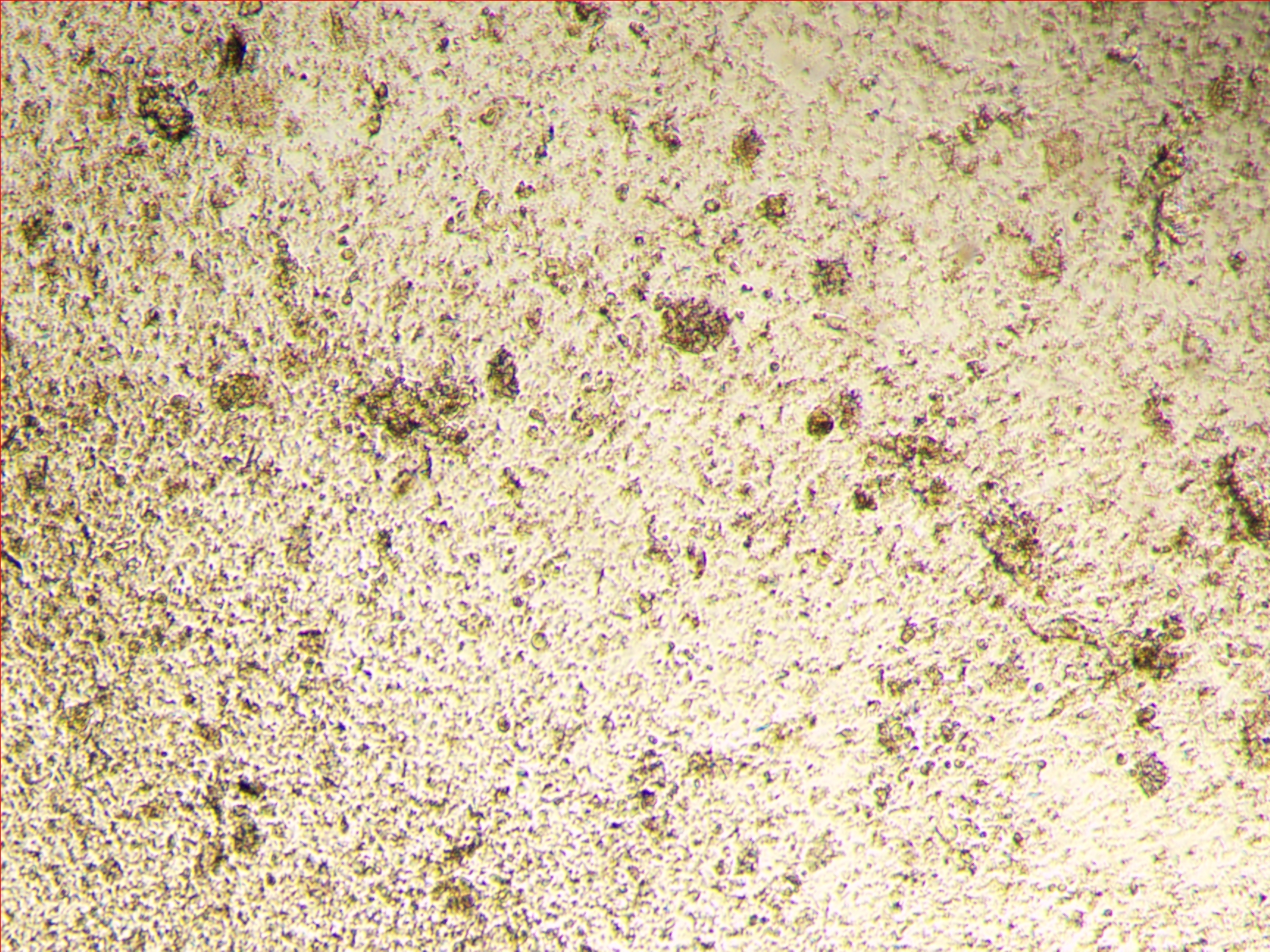

Supplement: Supplementary file 1 [file biology-15-00583-s001.zip › 100 mgL cholesterol-10×.jpg]

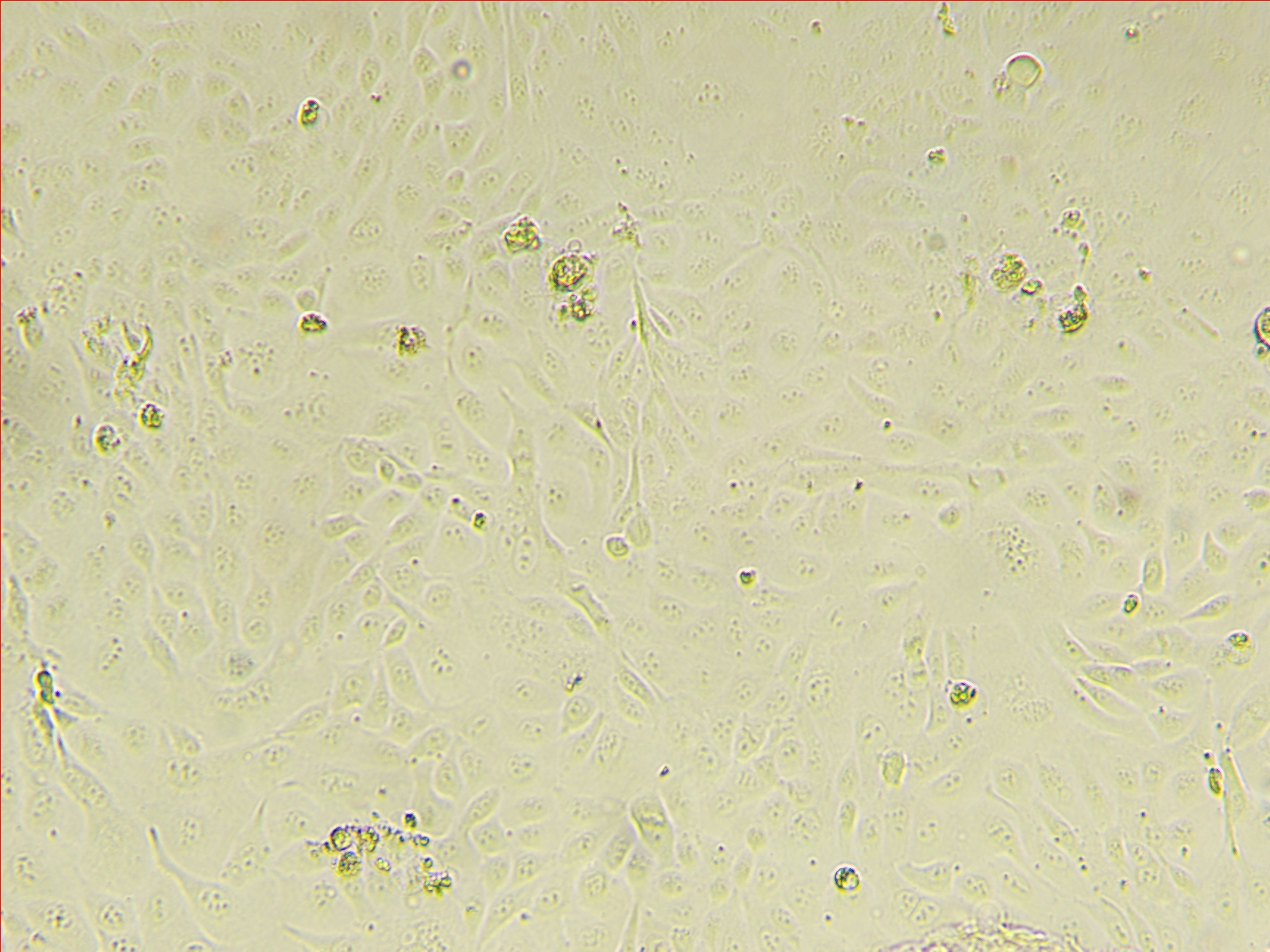

Supplement: Supplementary file 1 [file biology-15-00583-s001.zip › 20 mgL cholesterol-10×.jpg]

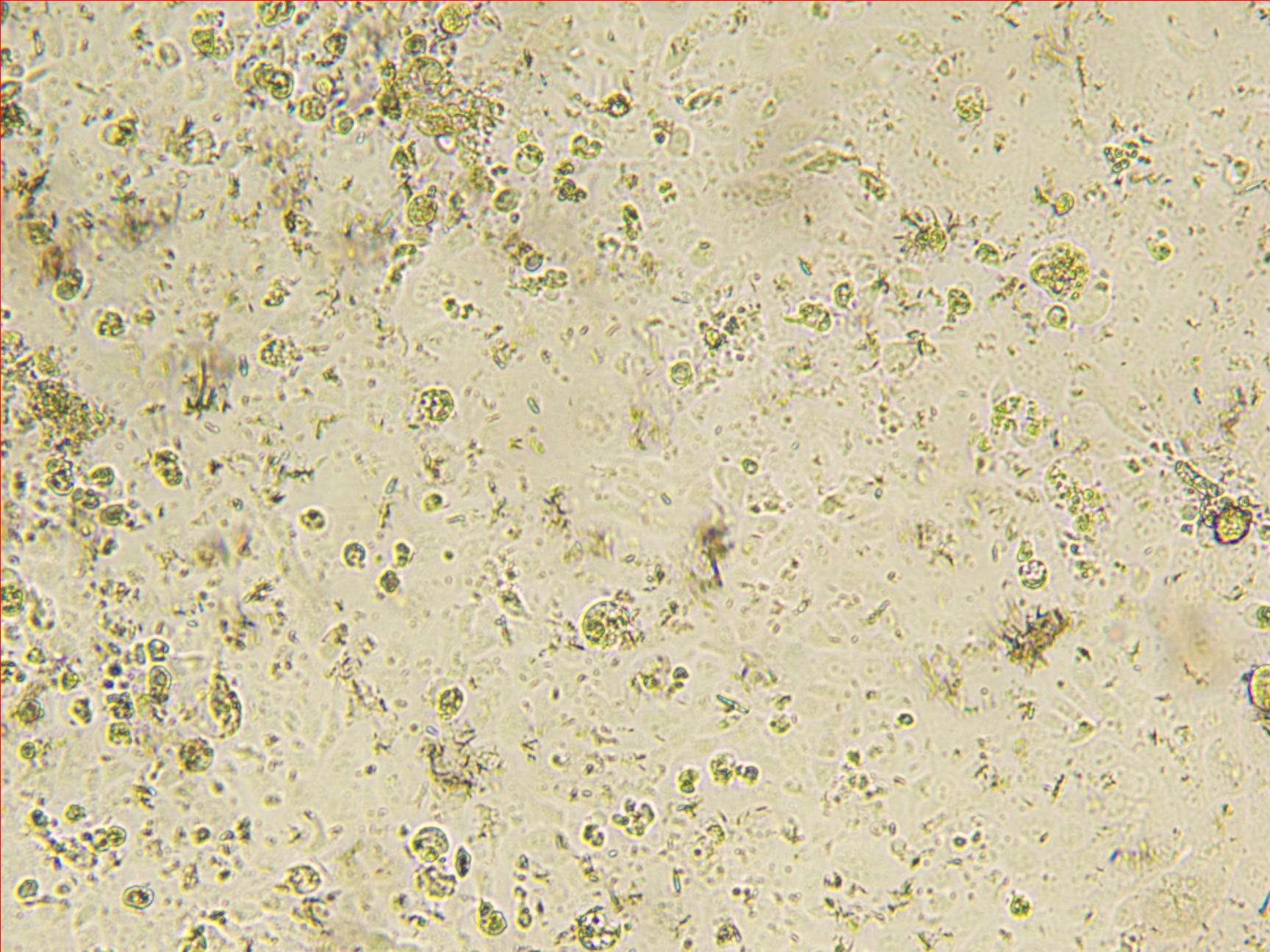

Supplement: Supplementary file 1 [file biology-15-00583-s001.zip › 30 mgL cholesterol-10×.jpg]

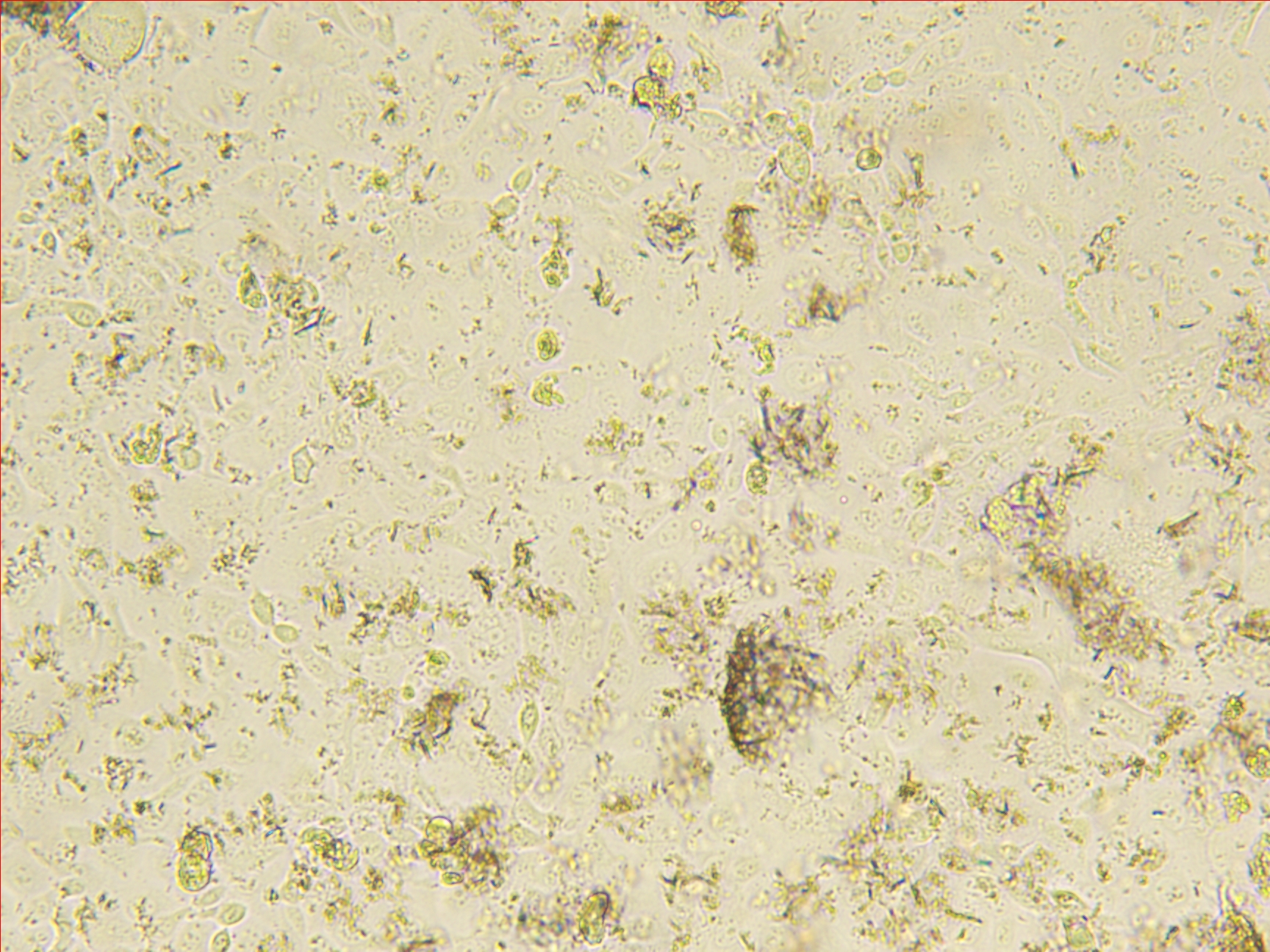

Supplement: Supplementary file 1 [file biology-15-00583-s001.zip › 40 mgL cholesterol-10×.jpg]

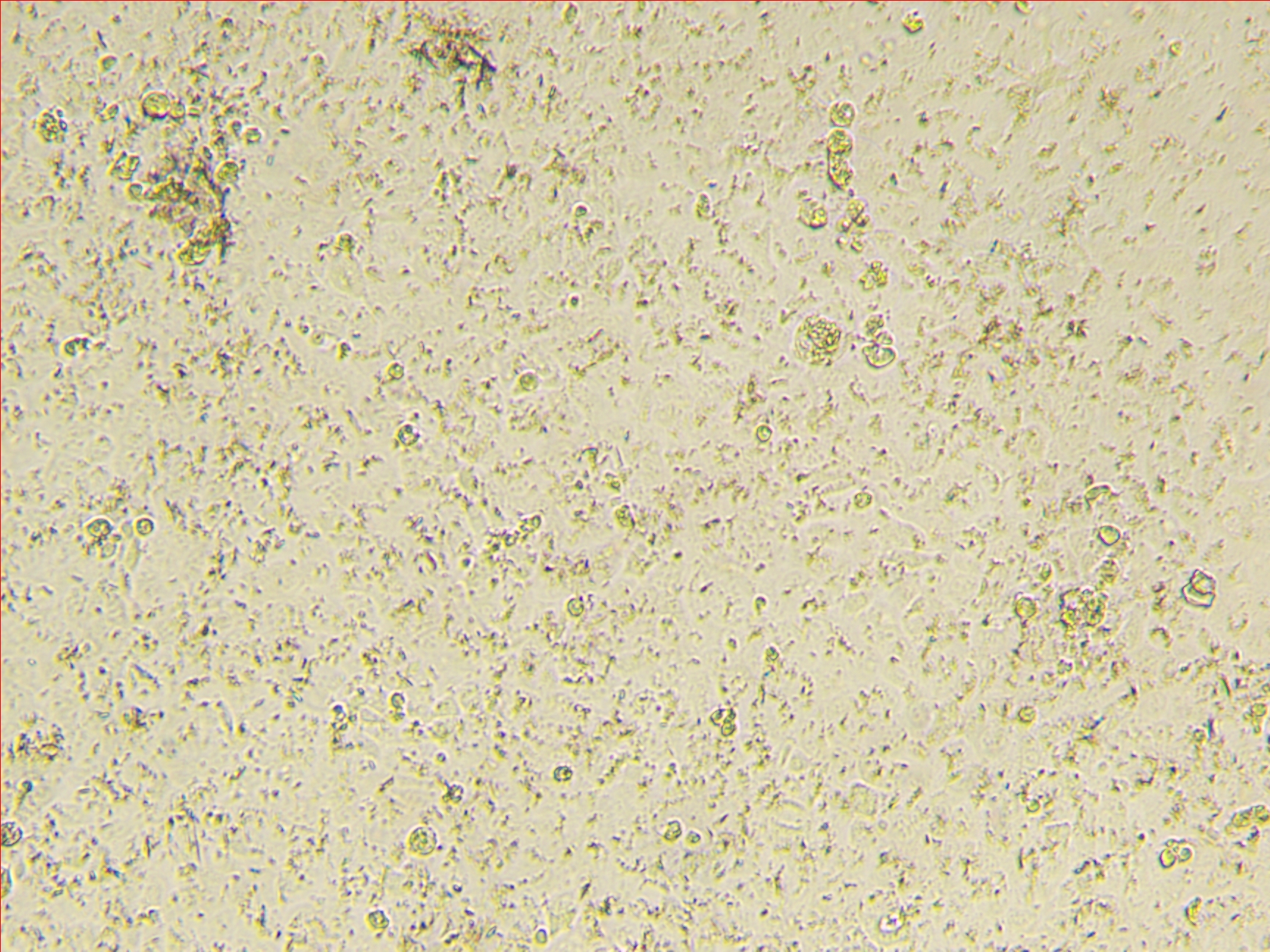

Supplement: Supplementary file 1 [file biology-15-00583-s001.zip › 50 mgL cholesterol-10×.jpg]

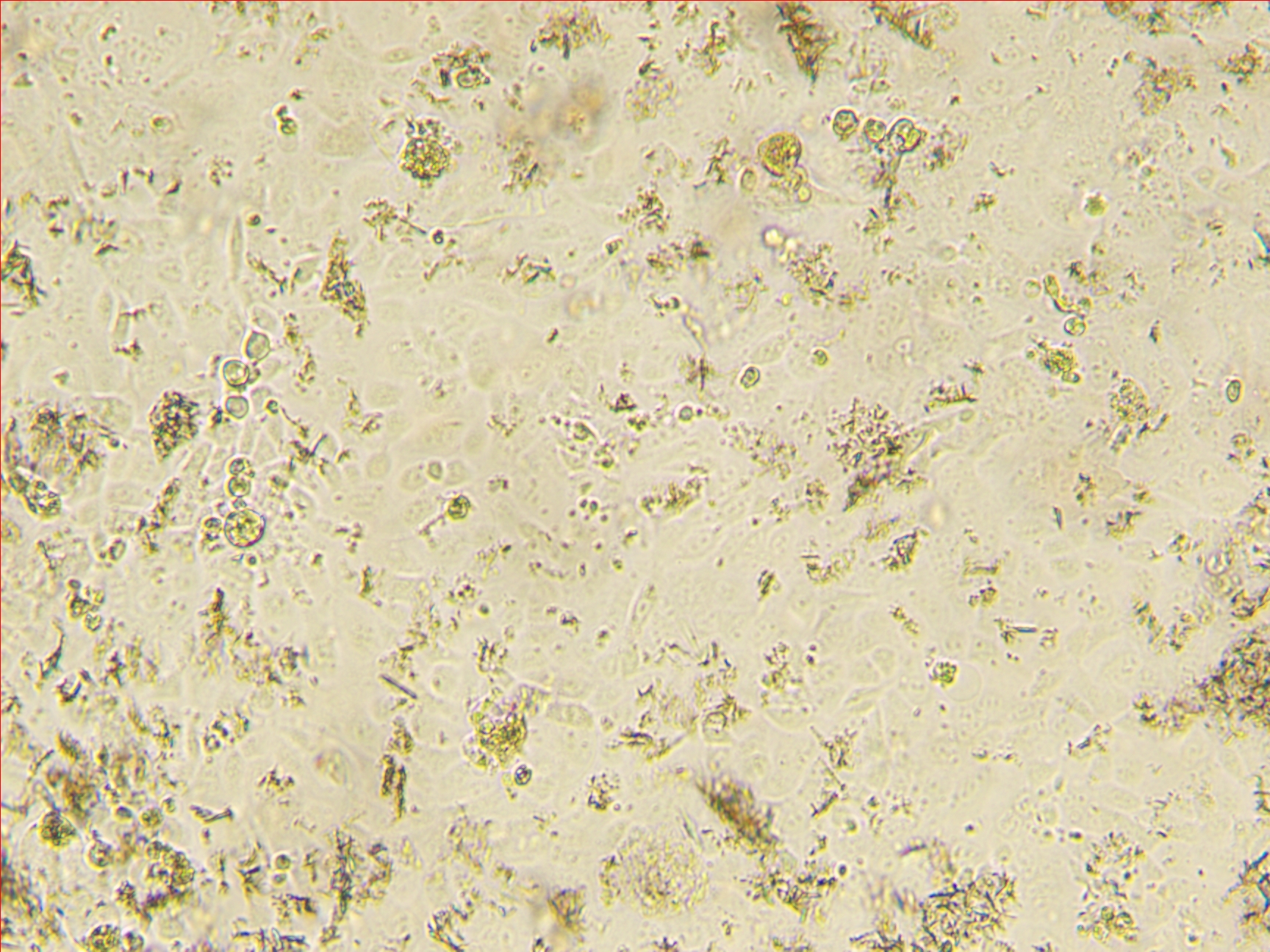

Supplement: Supplementary file 1 [file biology-15-00583-s001.zip › 60 mgL cholesterol-10×.jpg]

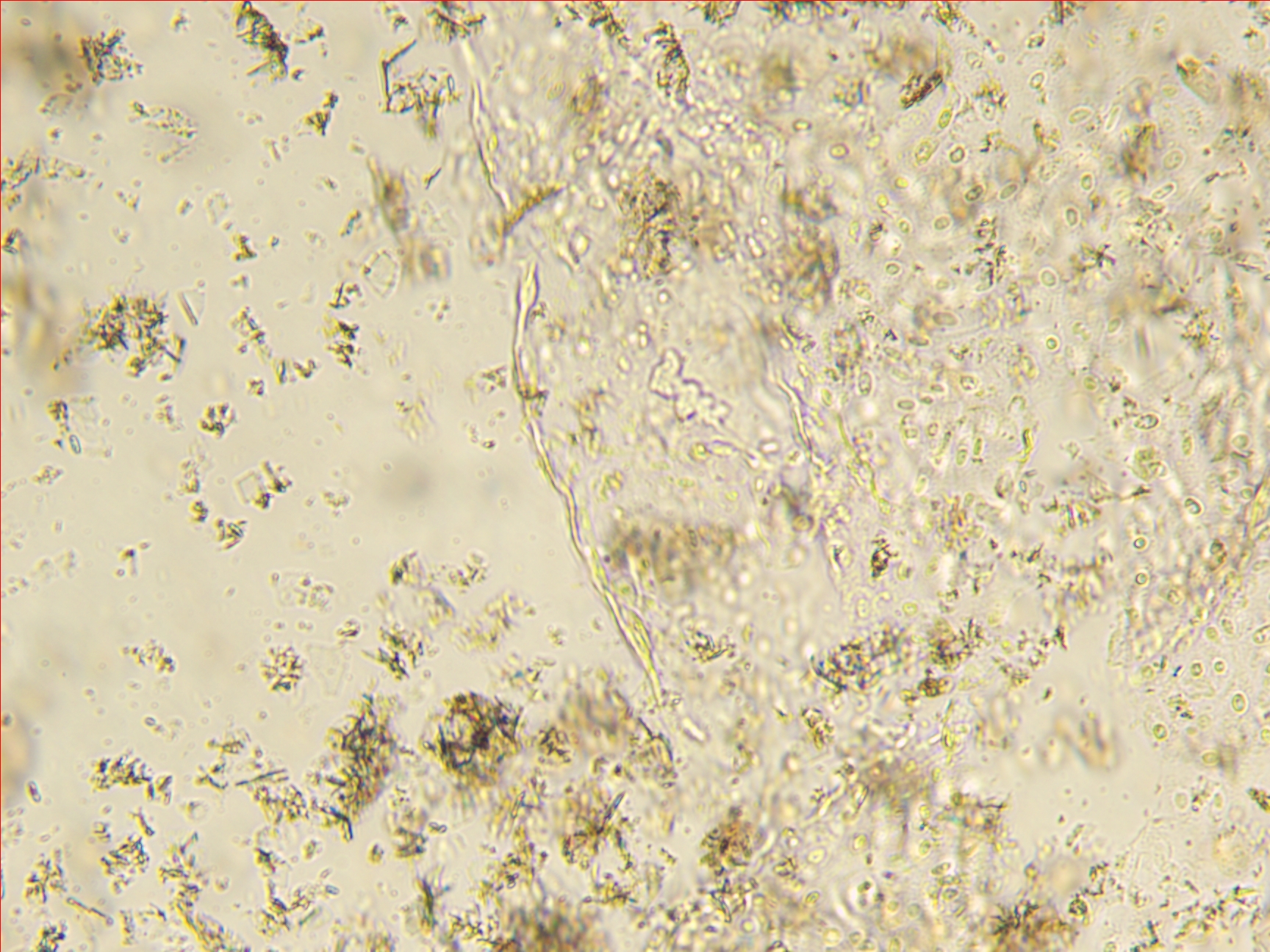

Supplement: Supplementary file 1 [file biology-15-00583-s001.zip › 70 mgL cholesterol-10×.jpg]

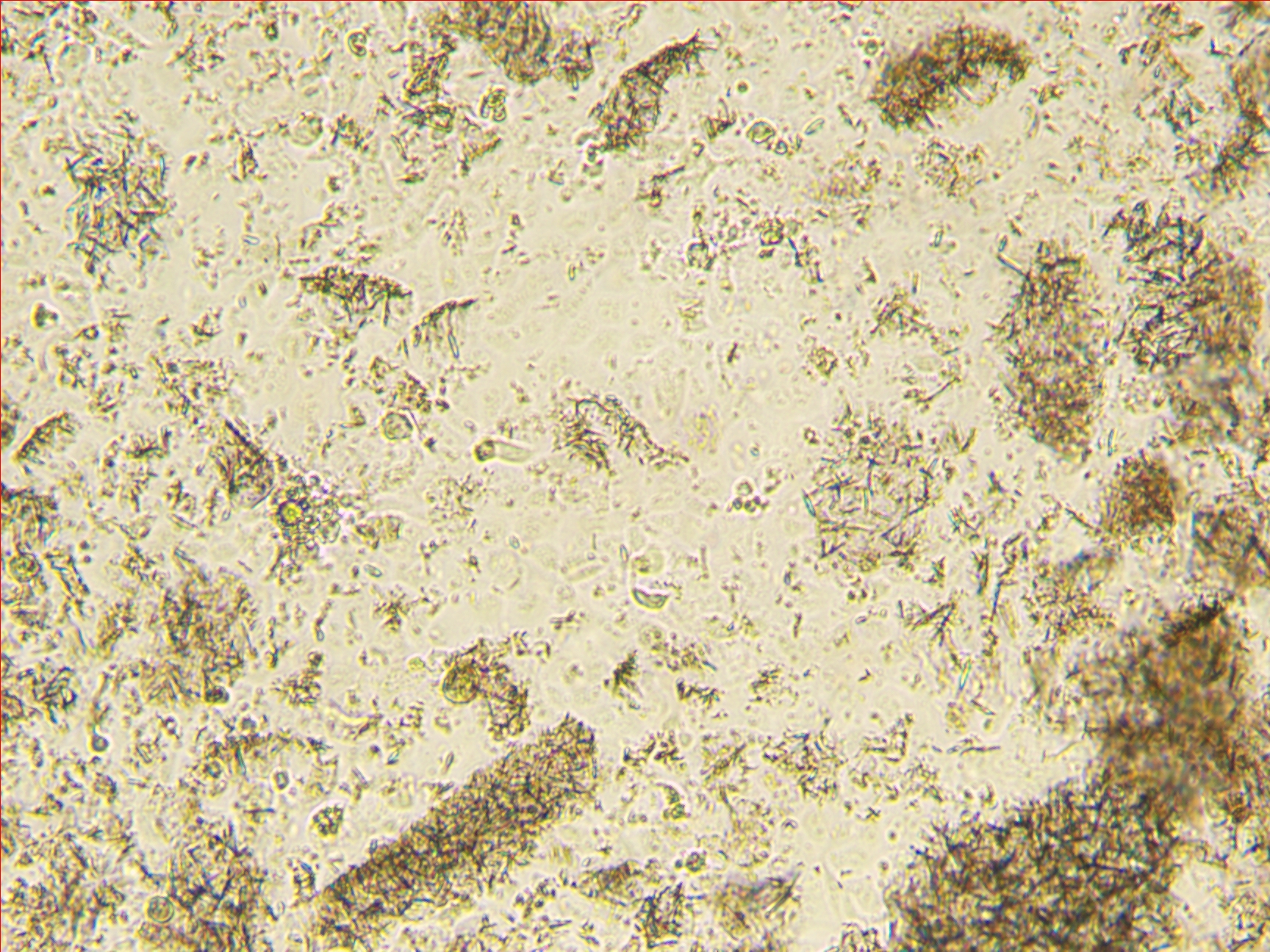

Supplement: Supplementary file 1 [file biology-15-00583-s001.zip › 80 mgL cholesterol-10×.jpg]

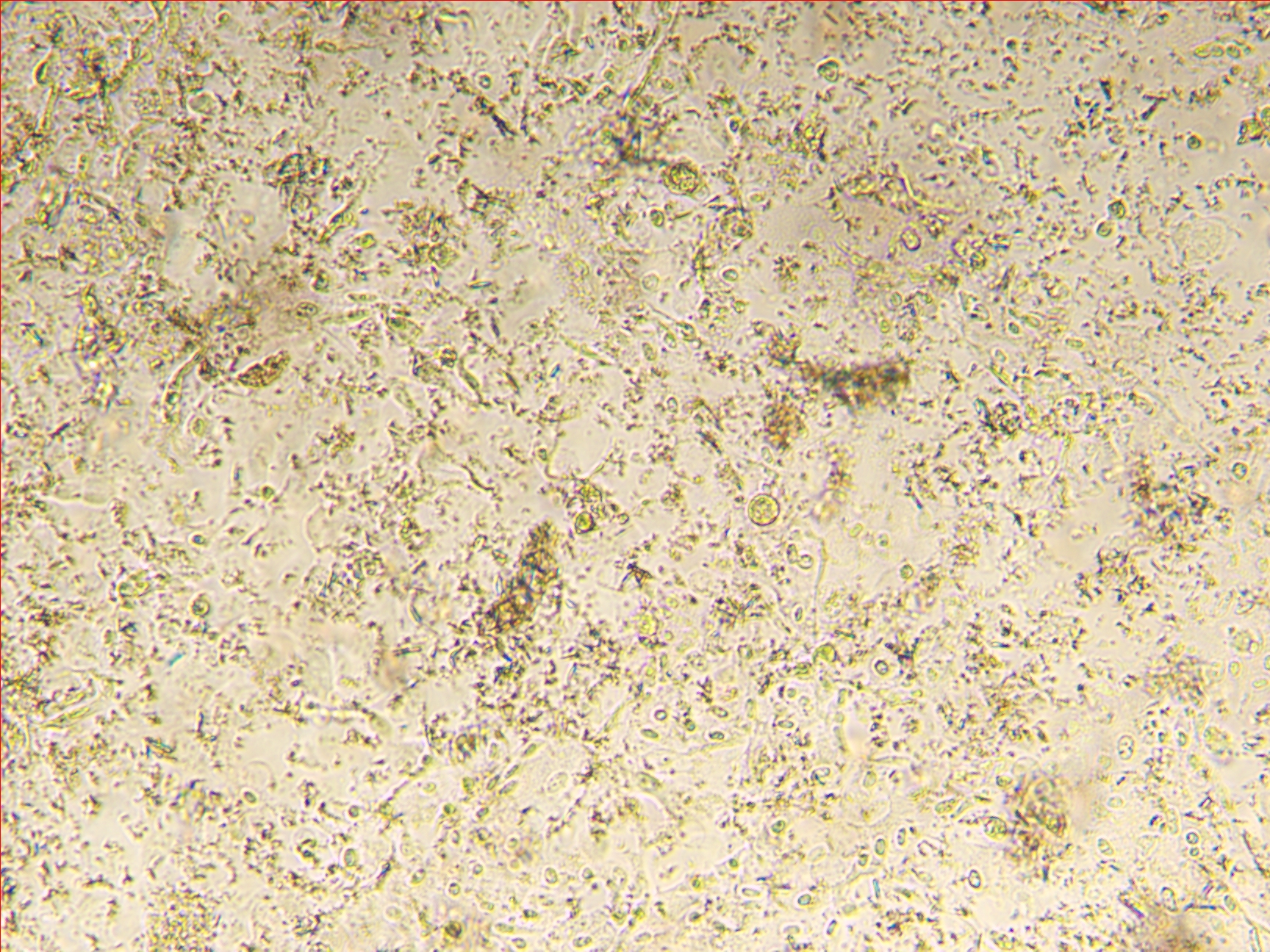

Supplement: Supplementary file 1 [file biology-15-00583-s001.zip › 90 mgL cholesterol-10×.jpg]

n\_genes\_by\_counts

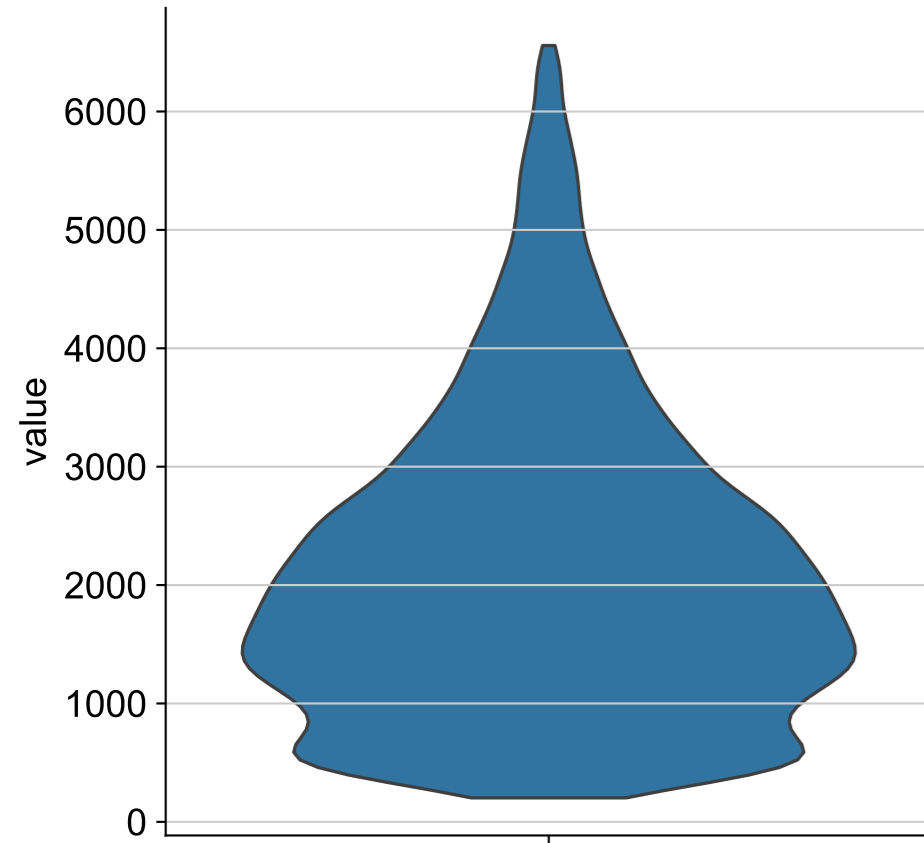

total\_counts

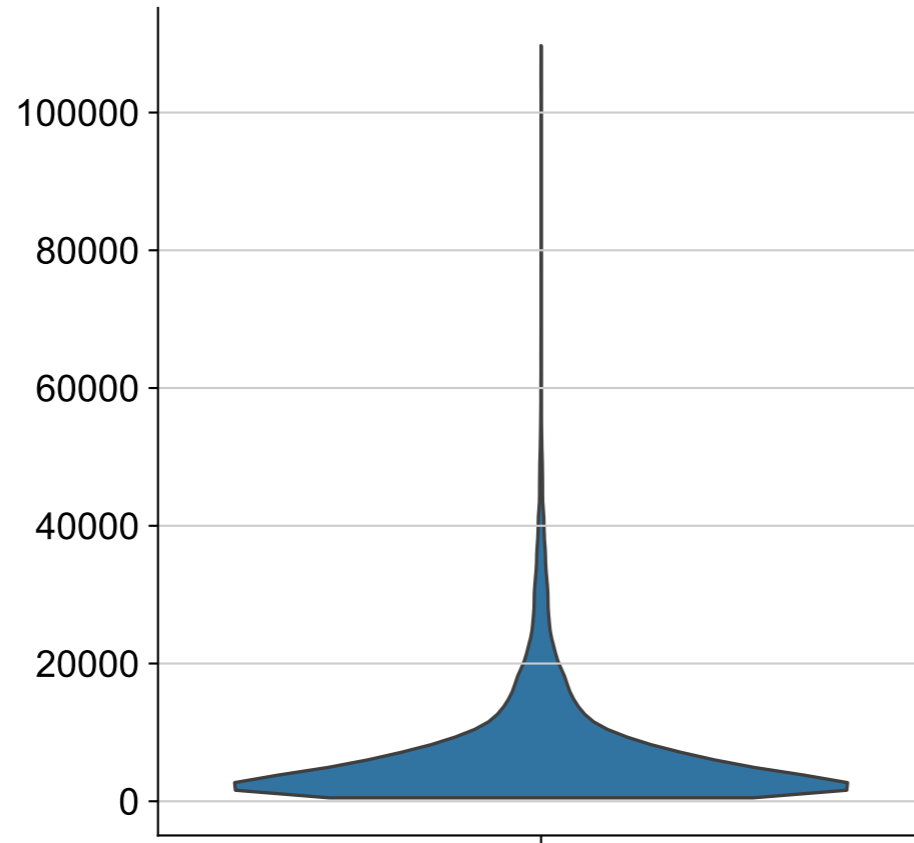

pct\_counts\_mt

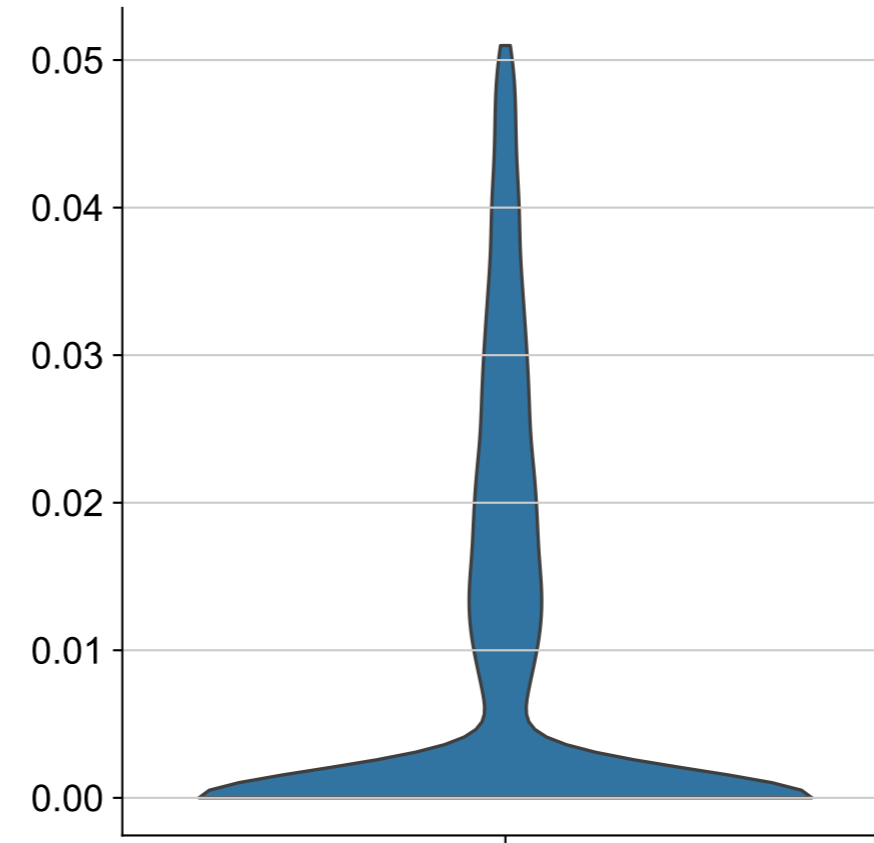

pct\_counts\_ribo

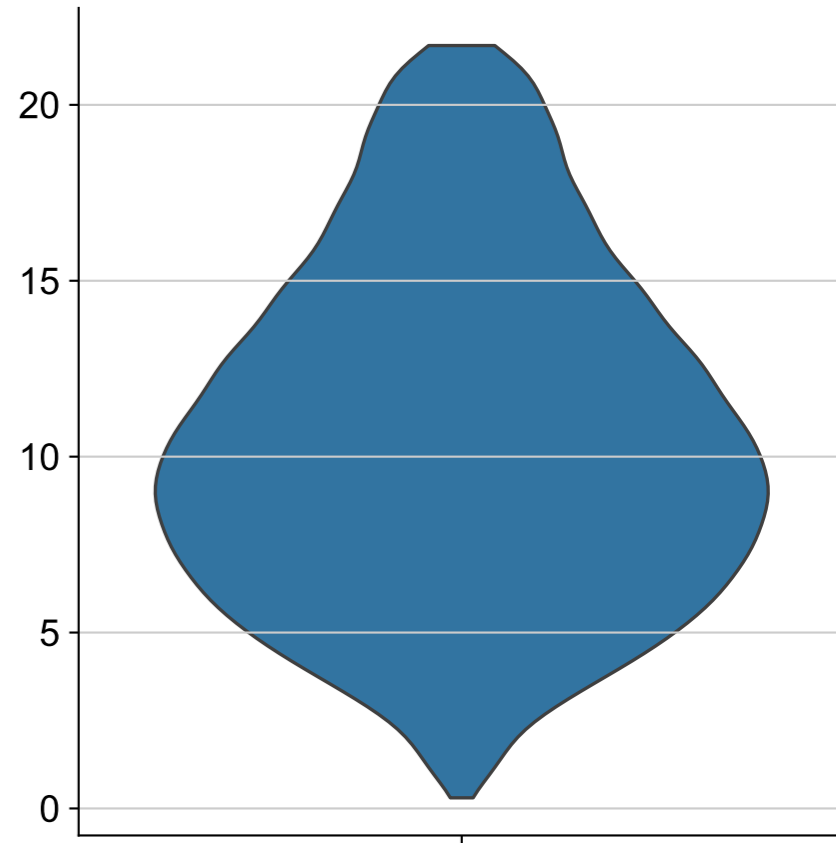

Supplement: Supplementary file 1 [file biology-15-00583-s001.zip › Figure S1 Pre-QC Violin Plots.pdf]

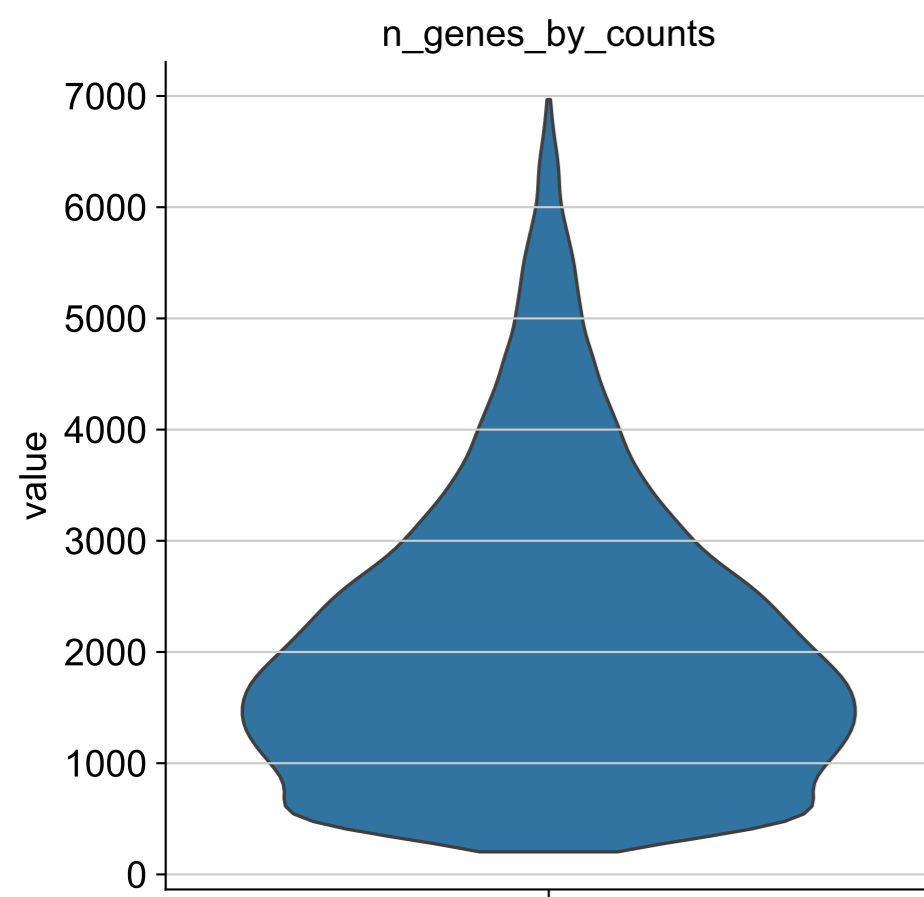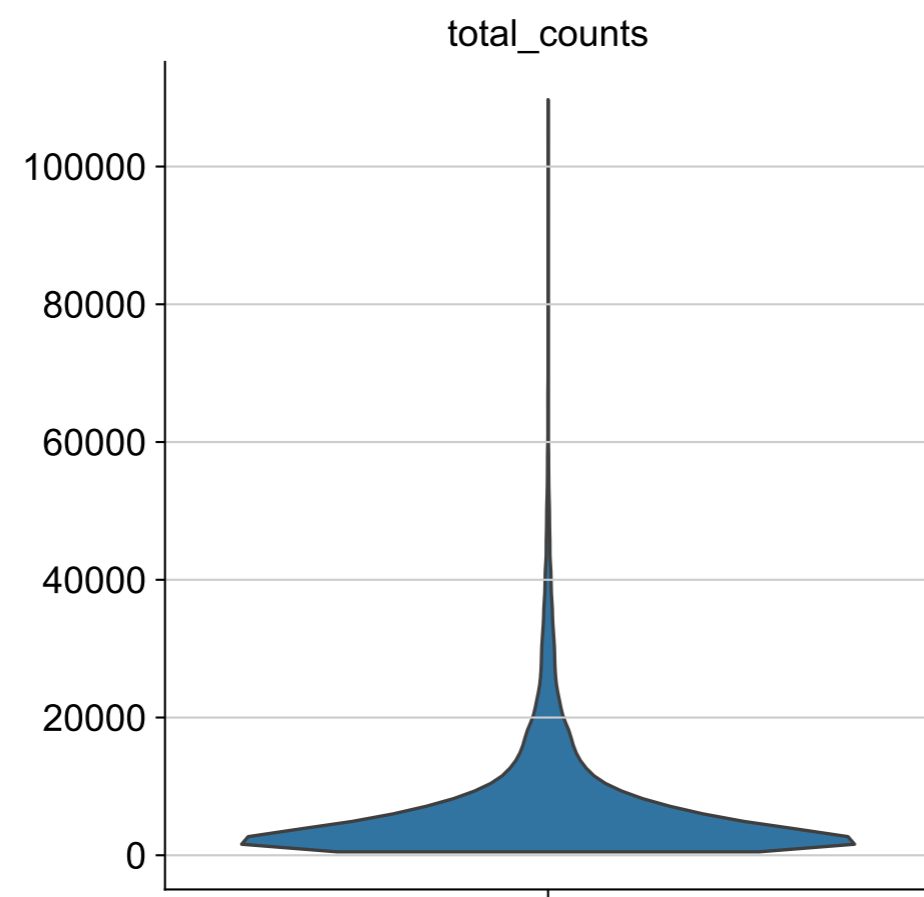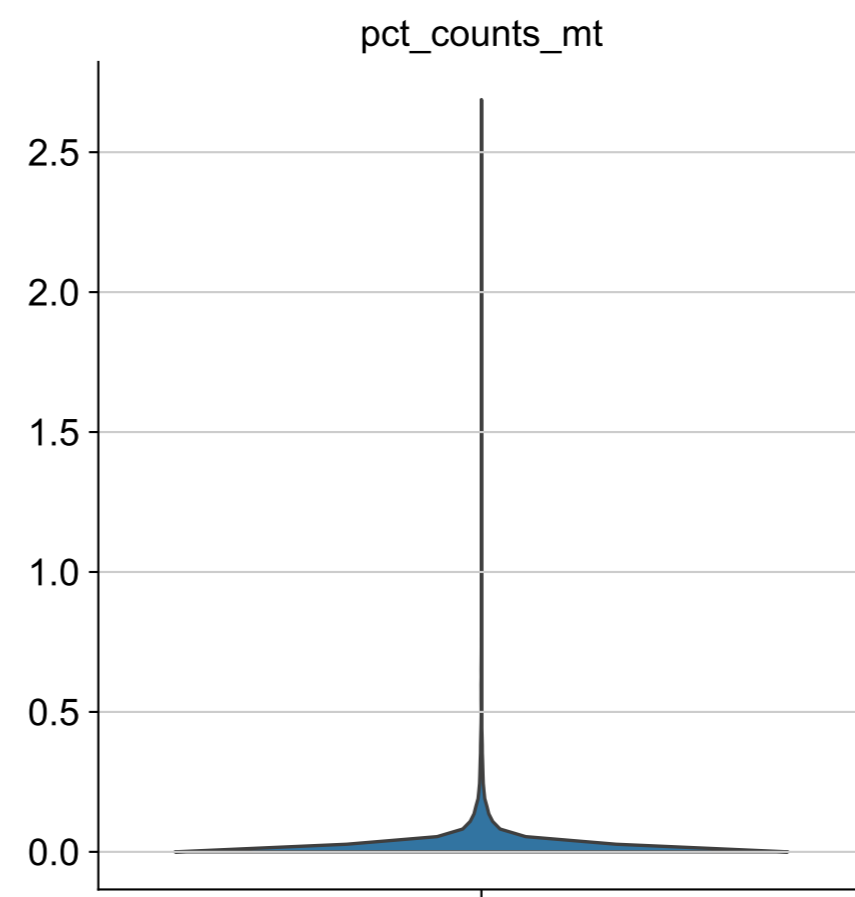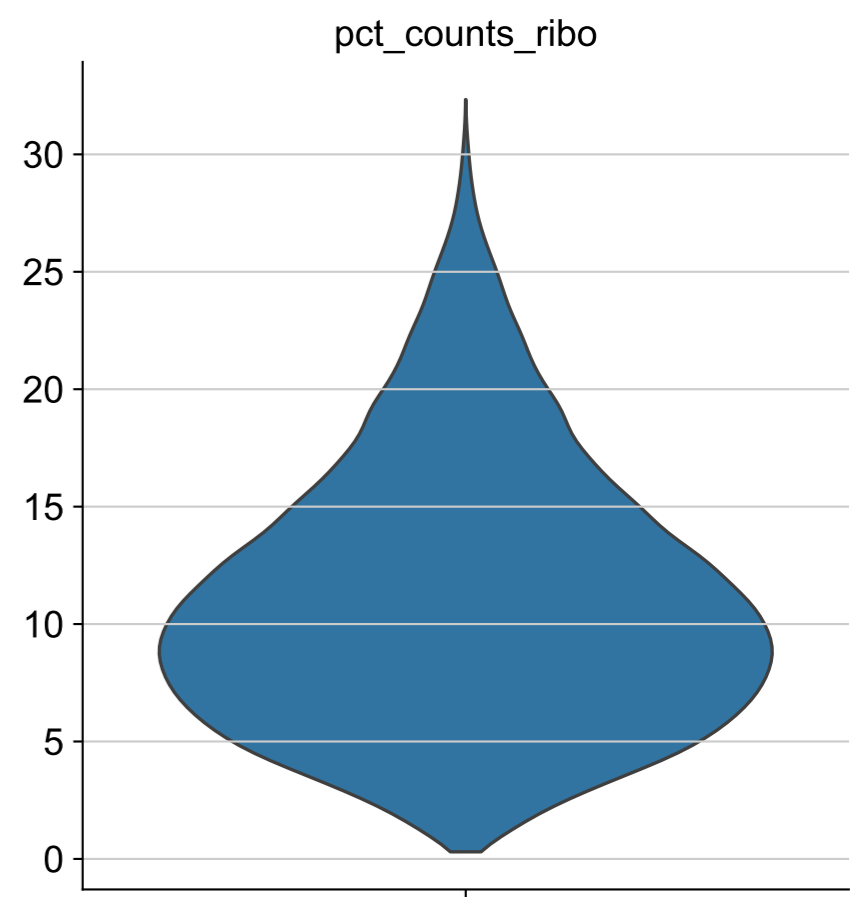

Supplement: Supplementary file 1 [file biology-15-00583-s001.zip › Figure S2 Post-QC Violin Plots.pdf]
